# The E3 ubiquitin ligase Sina regulates the assembly and disassembly of the synaptonemal complex in Drosophila females

**DOI:** 10.1371/journal.pgen.1008161

**Published:** 2019-05-20

**Authors:** Stacie E. Hughes, Elizabeth Hemenway, Fengli Guo, Kexi Yi, Zulin Yu, R. Scott Hawley

**Affiliations:** 1 Stowers Institute for Medical Research, Kansas City, Missouri, United States of America; 2 Department of Molecular and Integrative Physiology, University of Kansas Medical Center, Kansas City, Kansas, United States of America; The University of North Carolina at Chapel Hill, UNITED STATES

## Abstract

During early meiotic prophase, homologous chromosomes are connected along their entire lengths by a proteinaceous tripartite structure known as the synaptonemal complex (SC). Although the components that comprise the SC are predominantly studied in this canonical ribbon-like structure, they can also polymerize into repeated structures known as polycomplexes. We find that in Drosophila oocytes, the ability of SC components to assemble into canonical tripartite SC requires the E3 ubiquitin ligase Seven in absentia (Sina). In *sina* mutant oocytes, SC components assemble into large rod-like polycomplexes instead of proper SC. Thus, the wild-type Sina protein inhibits the polymerization of SC components, including those of the lateral element, into polycomplexes. These polycomplexes persist into meiotic stages when canonical SC has been disassembled, indicating that Sina also plays a role in controlling SC disassembly. Polycomplexes induced by loss-of-function *sina* mutations associate with centromeres, sites of double-strand breaks, and cohesins. Perhaps as a consequence of these associations, centromere clustering is defective and crossing over is reduced. These results suggest that while features of the polycomplexes can be recognized as SC by other components of the meiotic nucleus, polycomplexes nonetheless fail to execute core functions of canonical SC.

## Author summary

Mistakes that occur during meiotic chromosome segregation can lead to fetal death or various disorders in offspring, hence proper chromosome segregation is crucial. To ensure optimal execution of this process, crossing over must occur between homologous chromosomes. Crossovers form only in the presence of a large proteinaceous structure called the synaptonemal complex (SC), which forms between homologs during early meiosis. How SC components assemble in a controlled manner into a normal ribbon-like structure between homologs is poorly understood. In female fruit flies, mutations in *seven in absentia* (*sina*) cause SC components to self-assemble into large structures called polycomplexes, rather than into normal SC. Sina is a member of a family of E3 ubiquitin ligases, which are present in a number of organisms and target other proteins for degradation. We propose that there are proteins that promote the assembly of SC components into polycomplexes, and that Sina is required to degrade such proteins, allowing the normal assembly of SC components between homologous chromosomes.

## Introduction

The faithful segregation of chromosomes away from their homologs at the first meiotic division is the physical basis for Mendelian inheritance. In most organisms, chromosome segregation is achieved by recombination between paired homologs, a process that results in chiasmata and in the production of gametes bearing recombined chromosomes. Both the maintenance of homolog pairing and crossing over depend on the production of double-strand breaks (DSBs) in the context of the synaptonemal complex (SC). Meiotic DSBs are catalyzed by SPO11 homologs, and a subset of these DSBs are then converted into crossovers only in the presence of the SC [1,2]. By maturing into chiasmata, these crossovers serve to physically interlock homologs at metaphase I and thus ensure segregation.

**Fig 1:**
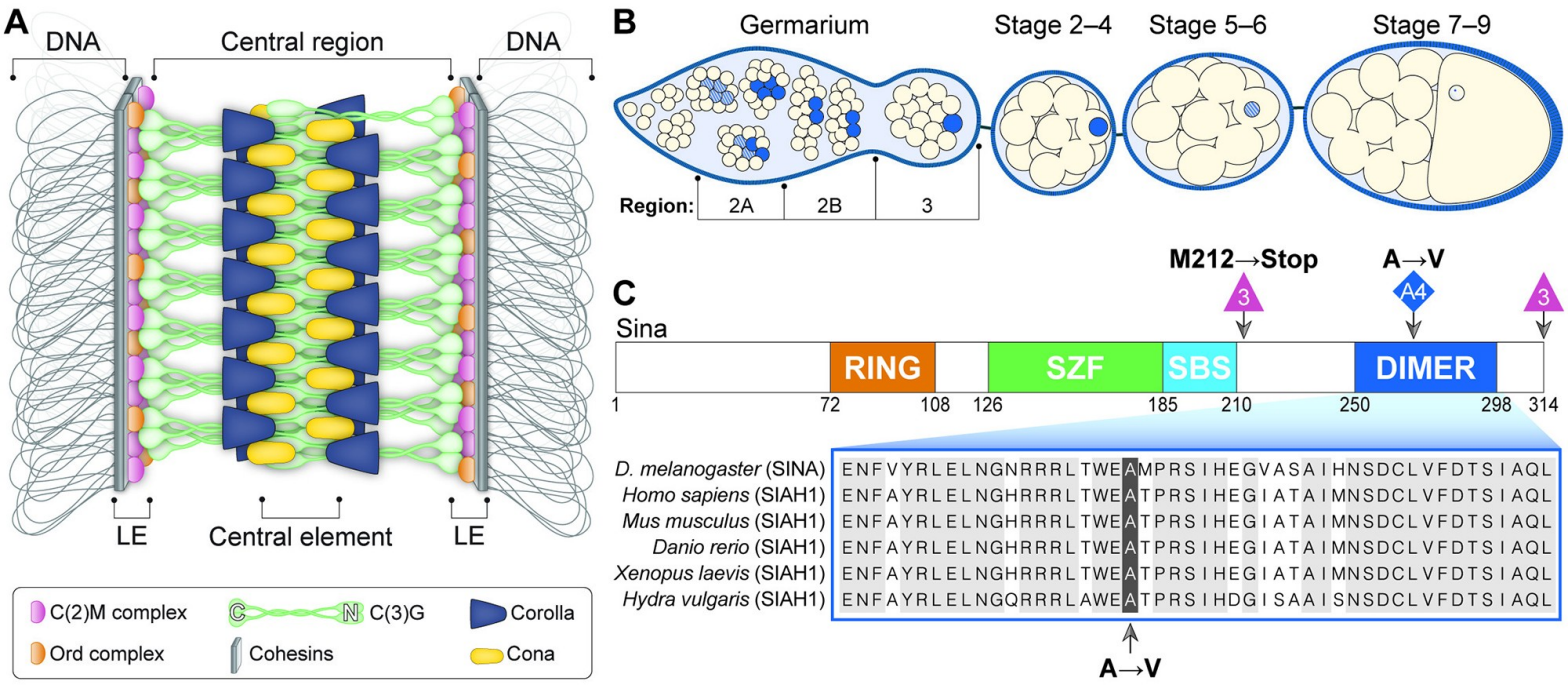
SC assembly in the Drosophila ovary. (A) Current model of the Drosophila SC along the euchromatin. (B) Schematic of the timing of SC assembly and disassembly in Drosophila females. At the initiation of meiosis (region 2A), SC components (blue) load along the chromosomes in up to four nuclei of the cyst. As the cyst progresses through the germarium (region 2B–3), the SC is progressively disassembled from all nuclei but the pro-oocyte. SC progressively disassembles from the chromosome arms in the oocyte at approximately stage 5–7 but persists at the centromeres for additional stages. (C) The *sina*^*A4*^ mutation affects a conserved amino acid in the dimerization domain of the Sina protein. The domains of the Sina protein are as described in [16]. The RING (Really Interesting New Gene) domain has E3 ligase catalytic activity, the SZF (SIAH-type zinc finger) domain includes a dual zinc-finger motif, the SBS (substrate-binding site) recognizes some targets, and the DIMER (dimerization) domain allows for formation of homo- and heterodimers of Sina family proteins. The location of the *sina*^*A4*^ mutation (highlighted) in the DIMER domain is shown in comparison to sequences from Siah1 homologs in other species (for full homology see [16]). The location of the deletion breakpoints and the resulting early stop of the *sina*^*3*^ mutation is also shown.

In *Drosophila melanogaster* females, as well as many other organisms, the SC assembles along each pair of homologous chromosomes during early pachytene [3,4,5]. Proper SC formation is required not only for chromosome synapsis, but also for the maturation of DSBs into crossovers [6,7,8,9]. The SC also plays a pivotal role in the clustering of centromeres in Drosophila females [10,11]. As analyzed by electron microscopy (EM), the SC has a tripartite zipper-like structure that is highly conserved across many organisms [5,12,13,14]. Electron dense lines, referred to as the lateral elements (LEs), associate along each of the homologous chromosomes. Lateral element components include the cohesin and cohesin-related proteins that connect the chromatin to the rest of the SC (Fig 1A). The structure between the LEs is called the central region, and the electron-dense line that runs down the center of the central region is referred to as the central element. While the amino acid sequences of SC proteins evolve rapidly between even closely related species [15], the basic tripartite structure of the SC is maintained from yeast to humans, indicating the SC’s structure is crucial for its function in meiosis [5].

A schematic of the current structural model of the Drosophila SC is shown in Fig 1A. The transverse filament protein Crossover Suppressor on 3 of Gowen (C(3)G) spans the distance between LEs [6]. C(3)G is thought to form homodimers with their C-terminal ends at the LE and their N-terminal ends at the central element [6,17]. The Corona (Cona) and Corolla proteins appear to help stabilize and/or assemble C(3)G since the loss of any of the three proteins leads to a loss of SC assembly [7,8].

SC components are assembled and disassembled in a highly controlled fashion (Fig 1B). However, the process by which they are loaded and unloaded from the chromosomes in the Drosophila ovary is poorly understood. Recent work in other organisms has demonstrated the importance of post-translational modifications in regulating the timing and pattern of SC assembly and disassembly [5]. These modifications, including phosphorylation, acetylation and sumoylation can be on the SC components themselves as well as on regulators of SC assembly [5,18,19,20,21].

When assembly of central region components along the chromosomes is disrupted (for example, when LE proteins are absent or when excess free SC components are present), aberrant SC-like structures called polycomplexes can form [22,23,24,25,26,27]. Polycomplexes have been observed in many organisms, and by EM they appear to be repeating layers of SC [12,22,23,26,28]. Immunofluorescence studies of these structures in yeast, worms and flies have demonstrated that the polycomplexes contain central region proteins [12,21,22,24,29,30,31,32]. Polycomplex-like structures have been observed in cultured mammalian somatic cells upon expression of only the major transverse filament protein, SYCP1 [33]. Polycomplexes have been observed both in the cytoplasm and within the nucleus, and many of the better-characterized examples of polycomplexes are not associated with chromatin [22,23,24]. The mechanisms that ensure SC proteins assemble between homologous chromosomes and thus block self-assembly into polycomplexes are not well understood in many organisms [23,27].

We show here that mutations in the gene encoding the E3 ubiquitin ligase *seven in absentia* (*sina*) result in the aberrant formation of numerous and large SC polycomplexes in *D*. *melanogaster* females, demonstrating that Sina is required to promote normal SC assembly (perhaps by blocking PC formation). These polycomplexes can persist into meiotic stages beyond those at which SC has been fully disassembled in wild-type oocytes, suggesting that normal disassembly of the SC also requires a functional Sina protein. While these polycomplexes can associate with chromatin at both centromeres and at sites of DSBs, they fail to maintain centromere clustering or promote wild-type levels of crossover formation. Intriguingly, lateral element and cohesin proteins appear to be recruited to many of the *sina* mutant-induced polycomplexes.

Previous work in the Drosophila eye as well as numerous studies of homologs in other organisms indicate Sina and its homologs function as E3 ubiquitin ligases that target proteins for degradation [34,35,36,37,38]. Therefore, it seems likely that Sina regulates the destruction of a protein crucial for directing proper SC assembly and maintenance along the arms of the chromosomes and that loss of functional Sina protein leads to uncontrolled assembly of SC components into polycomplex structures.

## Results

### A mutation in *sina* causes meiotic chromosome nondisjunction

In somatic tissues, Sina acts as an E3 ubiquitin ligase that targets specific proteins for degradation [34,35,36,37]. For example, in the Drosophila eye, Sina has been shown to be required for degradation of the transcription factor Tramtrack [36]. Null alleles of *sina* are reported to cause numerous phenotypes, including lethargy, short lifespan, eyes with missing R7 photoreceptors, and male and female sterility [39]. However, a role for *sina* in meiosis has not been reported.

A forward genetic screen in our laboratory for mutants with elevated levels of *X* chromosome meiotic nondisjunction produced a mutant, termed *A4*, which exhibited elevated meiotic nondisjunction. Deficiency mapping and sequencing identified *A4* as a new allele of *sina*, hereafter called *sina*^*A4*^. The *sina*^*A4*^ mutation changes the highly conserved amino acid at position 270 from an alanine to a valine in the predicted Sina protein (Fig 1C). Unlike null alleles of *sina*, which are female-sterile [39], *sina*^*A4*^ females (as well as *sina*^*A4*^*/sina*^*Df*^ females) are semi-fertile, demonstrating that *sina*^*A4*^ is a hypomorphic mutation (S1A Fig). Additionally, both *sina*^*A4*^ and *sina*^*A4*^*/sina*^*Df*^ flies survive the 10 days necessary for genetic assays while *sina* null flies are lethargic and short-lived [39]. The increase in fertility and hardiness of *sina*^*A4*^ females compared to *sina* null females makes this a more tractable background in which to study *sina*’s meiotic roles.

In nondisjunction assays, *sina*^*A4*^ mutant females carrying normal-sequence *X* chromosomes displayed 13.4% *X* and 6.9% *4*^*th*^ chromosome nondisjunction, compared to 0.4% *X* and 0.2% *4*^*th*^ chromosome nondisjunction in control flies (Table 1). Since *sina*^*A4*^ is a hypomorphic allele and semi-fertile in combination with *sina*^*Df*^ (S1A Fig), we next analyzed the amount of chromosome nondisjunction in *sina*^*A4*^*/sina*^*Df*^ females (Table 1). *sina*^*A4*^*/sina*^*Df*^ females showed a more severe phenotype, with *X* and *4th* chromosome nondisjunction levels increasing to 47.4% and 28.2%, respectively. This level of *X* chromosome nondisjunction indicates the *X* chromosomes are segregating nearly at random. An N-terminal FLAG-tagged overexpression wild-type *sina* construct (^*FLAG*^*sina*^*WT*^) rescued the *X* chromosome nondisjunction phenotype, as well as the defect in egg hatch rate from *sina*^*A4*^*/sina*^*Df*^ females, confirming that *sina* is indeed our gene of interest (S1A and S1B Fig).

**Table 1:** *X* and *4*^*th*^ chromosome nondisjunction frequency.

Gamete Type				
Maternal	Paternal	Wild type^a^	*sina*^*A4*^	*sina*^*A4*^/*sina*^*Df*^
*X;4*	*XY; 44*	1638	234	155
*X;4*	*0; 44*	1421	302	179
*X* NDJ^b^				
*0; 4*	*XY; 44*	4	10	48
*XX; 4*	*0; 44*	2	21	58
*4* NDJ				
*X; 0*	*XY; 44*	3	8	18
X; 0	*0; 44*	0	4	12
*X; 44*	*XY; 0*	0	1	23
*X; 44*	*0; 0*	3	7	13
*X; 4* NDJ				
*0; 0*	*XY; 44*	0	5	32
*XX; 44*	*0; 0*	0	4	21
*0; 44*	*XY; 0*	0	2	12
*XX; 0*	*0; 44*	0	1	9
Total Progeny		3065	599	580
Adjusted Progeny^c^		3077	642	760
% X NDJ		0.4	13.4**	47.4**
% 4 NDJ		0.2	6.9**	28.2**
% nullo-*X*		0.3	5.3	24.2
% diplo-*X*		0.1	8.1	23.2
% nullo-*4*		0.1	3.7	14.7
% diplo-*4*		0.1	3.1	13.4

^*a*^ Wild type = *y w; spa*^*pol*^. *sina*^*A4*^ = *y; sina*^*A4*^*; spa*^*pol*^. *sina*^*A4*^*/sina*^*Df*^ = *y w/y; sina*^*A4*^*/sina*^*Df*^*; spa*^*pol*^.

^*b*^ NDJ, nondisjunction

^*c*^ Adjusted Total is calculated to adjust for inviable progeny classes (see Methods).

**P<0.001 significantly different to wild type with the number of progeny scored. Statistical test described in [40].

### SC assembly is abnormal in *sina* mutants

To elucidate the cause of the chromosome nondisjunction in *sina*^*A4*^ and *sina*^*A4*^*/sina*^*Df*^ mutants, we examined the early steps of meiosis. Because similar high levels of chromosome nondisjunction are also observed in females mutant for SC components [6,7,8,9], we wondered whether SC formation was defective in *sina* mutants. In wild-type Drosophila females, central region components of the SC are first observed to load near the centromeres during the premeiotic mitotic divisions that produce the 16-cell interconnected cyst [41]. As the cyst enters meiosis in early pachytene (region 2A) of the germarium, the central region proteins rapidly load along the chromosome arms in up to four cells of the 16-cell cyst (Fig 1B), appearing as curved, ribbon-like tracks. As the cyst matures and moves through the germarium, the SC disassembles from three of the nuclei, leaving only the pro-oocyte nucleus with full-length SC by mid-pachytene (region 3) (reviewed in [3]). Full-length SC is maintained until approximately stage 5, when the SC along the chromosome arms begins to progressively disassemble. At approximately stage 7–8, SC components remain only at the centromeres.

**Fig 2:**
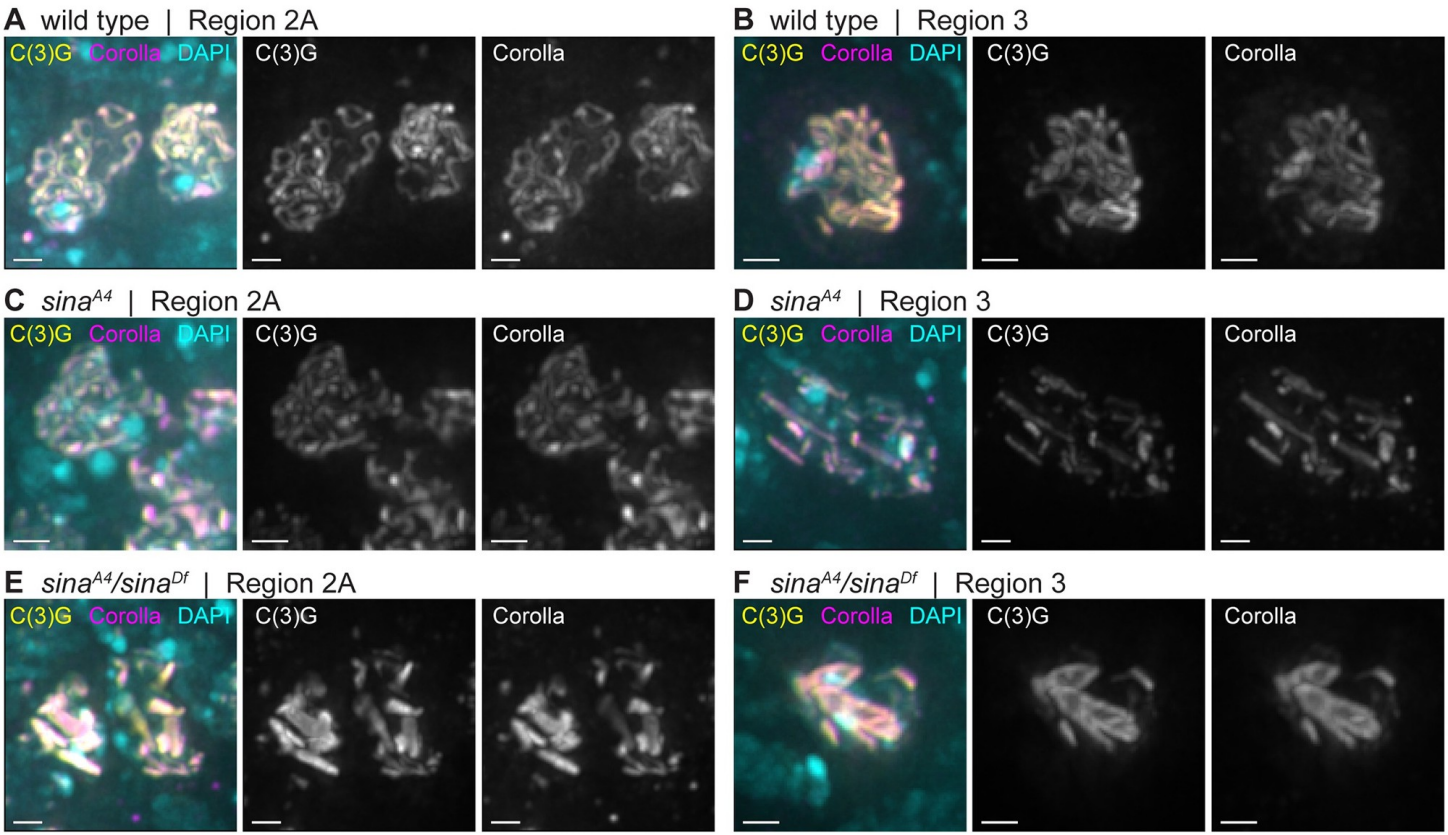
The central region components C(3)G and Corolla assemble into aberrant structures in *sina* mutants. Nuclei from (A, C, E) early pachytene (region 2A) and (B, D, F) mid-pachytene (region 3) are labeled with DAPI (cyan) and antibodies to Corolla (magenta) and the C-terminus of C(3)G (yellow). Scale bars, 1 μm. Images show projections of nuclei from larger *z*-stacks.

Deconvolution immunofluorescence microscopy using antibodies recognizing the central region proteins C(3)G and Corolla in *sina*^*A4*^ and *sina*^*A4*^*/sina*^*Df*^ ovaries revealed the presence of aberrant SC (Fig 2). In wild type, the SC forms as curved tracks between the chromosomes in early pachytene (region 2A) and the tracks of SC are present in the oocyte nucleus in mid-pachytene (region 3) (Fig 2A and 2B and S2A Fig). In *sina*^*A4*^ ovaries, the SC began to assemble with relatively normal timing, exhibiting tracks of SC in multiple nuclei in early pachytene (region 2A) (Fig 2C and S2B Fig). Full length SC tracks were observed in 46.5% (20/43) of *sina*^*A4*^ nuclei in early pachytene (region 2A). As the cysts progressed through the germarium, the SC lost its curved, track-like pattern and SC components began to form narrow rod-like structures (Table 2, Fig 2D and S2 Fig). In early pachytene (region 2A) 18.6% (8/43) of the nuclei displayed a combination of tracks and rod-like structures and 34.9% (15/43) of the nuclei contained only these rod-like structures. By early/mid-pachytene (region 2B) and mid-pachytene (region 3), 72.2% (13/18) and 87.5% (14/16) of nuclei respectively, had no clear SC tracks visible among the rod-like structures, and the remaining nuclei had partial pieces of track-like SC and polycomplexes (Fig 2D and S2B Fig). As the cysts budded from the germaria into the vitellarium at mid-prophase (stages 2–9), *sina*^*A4*^ oocyte nuclei retained the abnormal SC structures in 85.7% (12/14) of nuclei with 5 nuclei also containing fragments of track-like SC. In the remaining two nuclei only fragmented SC tracks were observed.

**Table 2:** Quantification of the number of polycomplexes per nucleus by stage.

Genotype	2A^a^	N	2B^a^	N	3^a^	N	St.2-9^a^	N
*sina*^*A4*^	7.7 (3.1)	23	8.2 (1.9)	18	8.6 (1.8)	16	5.6 (2.4)	12
*sina*^*A4*^*/sina*^*Df*^	3.8 (1.8)	29	5.0 (1.8)	22	5.4 (1.9)	13	5.4 (1.9)	25
*c(2)M; sina*^*A4*^	1.5 (1.1)	31	1.8 (1.1)	14	1.3 (0.5)	11	1.1 (0.4)	21
*c(2)M; sina*^*A4*^*/sina*^*Df*^	1.3 (0.7)	38	1.3 (0.5)	19	1.2 (0.4)	18	1.0 (0.0)	18

^*a*^ Standard deviation in parentheses.

Consistent with the stronger meiotic nondisjunction phenotype, *sina*^*A4*^*/sina*^*Df*^ females showed an exacerbated SC phenotype (Fig 2E and 2F), including fewer SC tracks and earlier polycomplex formation. We observed very small rod-like SC structures in the premeiotic region 1 where only foci of SC components are observed in wild type (S2D and S2E Fig). By early pachytene (region 2A), the SC had already assembled into aberrant structures in *sina*^*A4*^*/sina*^*Df*^ ovaries with 100% (29/29) of nuclei containing polycomplexes (Fig 2E). In early pachytene (region 2A) 58.6% (17/ 29) of the nuclei had SC tracks of various lengths along with the polycomplexes. The number of nuclei with only polycomplexes then increased by mid-pachytene (region 3) (Fig 2F), but a least one small SC track-like structure could still be observed with the polycomplexes in 31.8% (7/22) of early/mid-pachytene (region 2B), 23.1% (3/13) mid-pachytene (region 3), and 20.0% (5/25) mid-prophase (stages 2–9) nuclei. It is important to note that *sina*^*A4*^ homozygotes had a less severe defect than *sina*^*A4*^/*sina*^*Df*^ females, with the presence of nuclei with long curved SC tracks in early pachytene (region 2A) and less severe chromosome nondisjunction (Table 1), which supports our earlier assessment that the *sina*^*A4*^ mutation is a hypomorphic allele.

For reasons more fully described below, we have concluded that these aberrant SC structures are best classified as polycomplexes. In early pachytene (region 2A) *sina*^*A4*^ ovaries had an average of 7.7 polycomplexes per nucleus, but the number ranged from 1–15 polycomplexes (Table 2). A similar average number of polycomplexes could be observed in *sina*^*A4*^ ovaries through mid-pachytene (region 3), but then the average number decreased to 5.6 polycomplexes in mid-prophase (stage 2–9) oocytes (Table 2). The *sina*^*A4*^*/sina*^*Df*^ germaria displayed a lower average number of polycomplexes compared to *sina*^*A4*^ homozygotes with only 3.8 polycomplexes per nucleus in early pachytene (region 2A) (Table 2) with a range of 1–8 polycomplexes per nucleus. However, the average number of polycomplexes per nucleus in *sina*^*A4*^*/sina*^*Df*^ germaria increased to 5.0 in early/mid-pachytene (region 2B) and remained similar through mid-prophase (stages 2–9) (Table 2).

**Fig 3:**
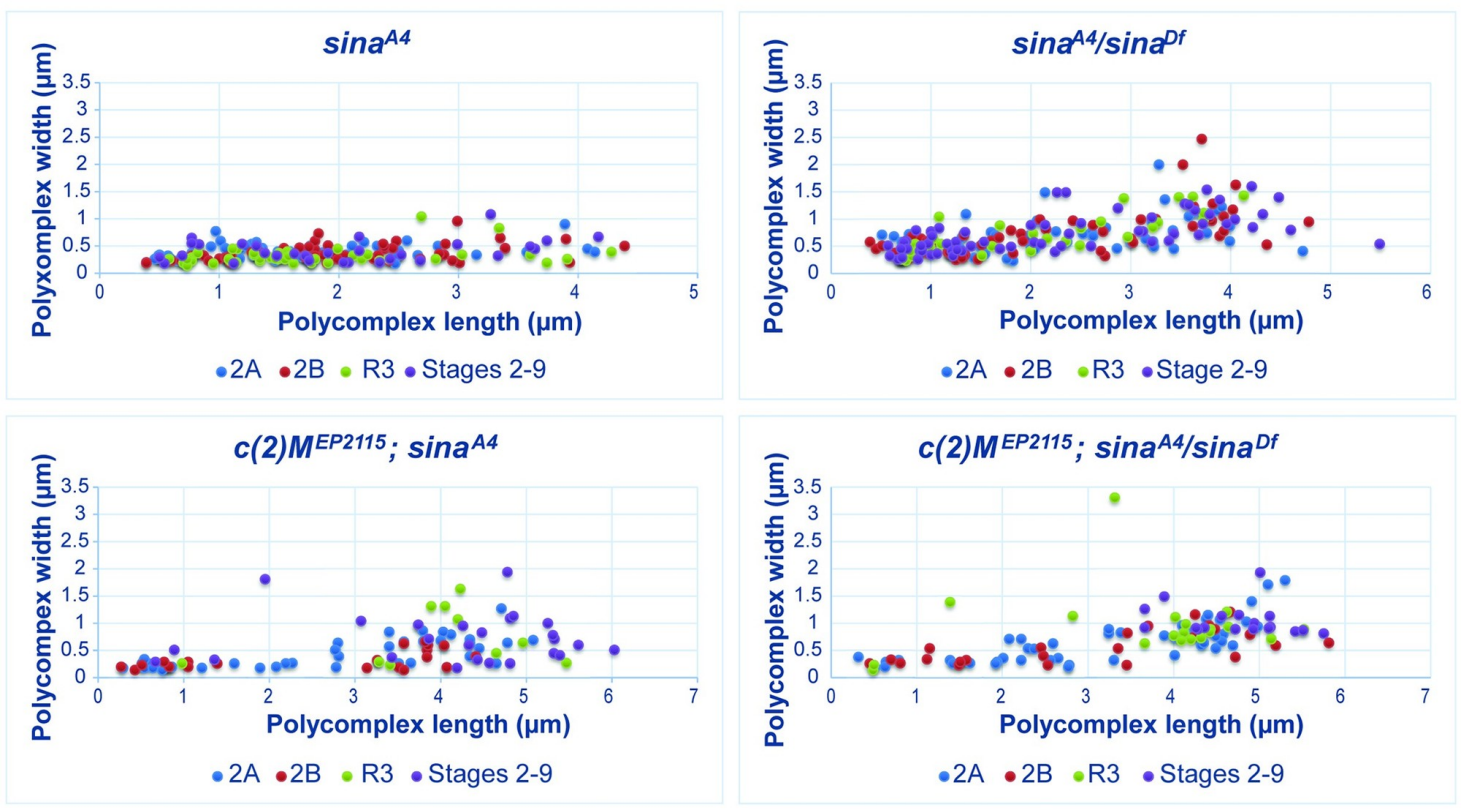
The length and width of polycomplexes in *sina* mutants. The measurements of the length and width of polycomplexes from *sina*^*A4*^, *sina*^*A4*^*/sina*^*Df*^, *c(2)M*^*EP2115*^; *sina*^*A4*^, and *c(2)M*^*EP2115*^; *sina*^*A4*^*/sina*^*Df*^ females. Colored dots show the measurements of the polycomplexes at early pachytene (region 2A) in blue, early/mid-pachytene (region 2B) in red, mid-pachytene (region 3) in green, and mid-prophase (stages 2–9) in purple. See S3 Fig for graphs separated by stage. Measurements are in microns.

While *sina*^*A4*^/*sina*^*Df*^ nuclei had a lower average number of polycomplexes than *sina*^*A4*^ homozygotes, the polycomplexes displayed more variability in width and had a greater average width than were seen in *sina*^*A4*^ homozygotes (Table 3, Fig 3, S3 Fig). For example, at early pachytene (region 2A) *sina*^*A4*^ had an average polycomplex width of 0.39 μm versus 0.69 μm for *sina*^*A4*^/*sina*^*Df*^ polycomplexes. The polycomplexes in *sina*^*A4*^ oocytes varied greatly in length, ranging from 0.20–4.28 μm but showed a relatively narrow range of widths with most polycomplexes less than 1 micron (Table 3, Fig 3, S3 Fig). Fig 2D illustrates this uniformity of width but not length of the polycomplexes, most of which were similar in appearance to rods.

**Table 3:** Average length and width of polycomplexes by stage.

	2A		2B		3		St.2-9	
Genotype^a^	Length^b^	Width^b^	Length^b^	Width^b^	Length^b^	Width^b^	Length^b^	Width^b^
*sina*^*A4*^	1.69 (0.89)^c^	0.39 (0.13)	1.96 (0.96)	0.37 (0.15)	1.76 (0.94)	0.32 (0.16)	2.02 (1.03)	0.41 (0.20)
*sina*^*A4*^*/sina*^*Df*^	2.17 (1.15)	0.69 (0.32)	2.12 (1.26)	0.71 (0.42)	1.99 (1.11)	0.71 (0.35)	2.24 (1.32)	0.73 (0.35)
*c(2)M; sina*^*A4*^	2.75 (1.53)	0.45 (0.27)	2.57 (1.52)	0.33 (0.17)	3.90 (1.16)	0.77 (0.51)	4.11 (1.56)	0.72 (0.51)
*c(2)M; sina*^*A4*^*/sina*^*Df*^	3.12 (1.44)	0.64 (0.38)	3.04 (1.63)	0.57 (0.31)	3.03 (1.52)	0.99 (0.85)	4.70 (0.66)	1.05 (0.30)

^*a*^ N values for *sina*^*A4*^ in order by stage were 62, 56, 44, and 30 polycomplexes measured. N values for *sina*^*A4*^*/sina*^*Df*^ were 61, 57, 32 and 64 polycomplexes measured. N values for *c(2)M*^*(EP2115)*^*; sina*^*A4*^ were 37, 25, 11, 17 polycomplexes measured. N values for *c(2)M*^*(EP2115)*^*; sina*^*A4*^*/sina*^*Df*^ was 47, 24, 11, and 15 polcomplexes measured.

^b^ Measurements in microns.

^*c*^ Standard deviation in parentheses.

In *sina*^*A4*^/*sina*^*Df*^ ovaries the average width of the polycomplexes was greater than was observed in *sina*^*A4*^ homozygotes at all stages (Table 3, Fig 3, S3 Fig). A subset of the polycomplexes showed a similar width of around 0.5 microns, but polycomplexes that were much wider could also be observed (Fig 3, S3 Fig). The average length of the polycomplexes in *sina*^*A4*^/*sina*^*Df*^ nuclei remained similar throughout the stages, with a length of 2.17 μm at early pachytene, but as was observed for polycomplexes in *sina*^*A4*^ homozygotes there was a large degree of variability. The maximum polycomplex length increased to 5.49 μm in *sina*^*A4*^/*sina*^*Df*^ ovaries versus 4.28 μm in *sina*^*A4*^ovaries (Table 3, Fig 3, S3 Fig). Rather than predominantly rod-like structures some of the larger polycomplexes tapered at one or both ends in *sina*^*A4*^/*sina*^*Df*^ nuclei, such as shown in Fig 2E and 2F.

We find the relatively small variation in width, but not length of the polycomplexes in *sina*^*A4*^ nuclei intriguing. Fig 3 shows the that width of the majority of the polycomplexes are 0.5 μm or a narrower while the length ranges over several microns. In *sina*^*A4*^/*sina*^*Df*^ ovaries similar polycomplex widths predominate for polycomplexes under 2 μm in length, but as the polycomplexes increase in length the variability in width increases (Fig 3 and S3 Fig). As the *sina*^*A4*^ allele is a hypomorph, the mutated Sina protein may retain enough function to constrain the polycomplex width in *sina*^*A4*^ homozygotes; but reducing the mutated protein dose by half in *sina*^*A4*^/*sina*^*Df*^ nuclei may loosen this constraint.

Polycomplexes were also observed in females carrying the *sina*^*3*^ null allele in trans to either *sina*^*Df*^ or *sina*^*A4*^ [42] (S4A–S4C Fig), as well as in females bearing the *sina*^*P21*^ allele in trans to *sina*^*Df*^ [43,44] (S4D Fig). High chromosome nondisjunction was also observed for *sina*^*A4*^*/sina*^*3*^ females (S1 Table) with 52.4% *X* and 34.7% *4*^*th*^ chromosome nondisjunction. As the *sina*^*3*^ allele only affects the *sina* coding sequence [39] (Fig 1C), the presence of polycomplexes in *sina*^*A4*^*/sina*^*3*^ ovaries (S4B and S4C Fig) further supports our conclusion that the *sina* ^*A4*^ mutation is responsible for the polycomplex phenotype in *sina* ^*A4*^ homozygotes. We noted earlier that overexpression of a ^*FLAG*^*sina*^*WT*^ construct rescued the chromosome nondisjunction of *sina*^*A4*^/*sina*^*Df*^ females, and overexpression of the same construct rescued the aberrant SC phenotypes (S1C Fig). Thus, polycomplex formation appears to be a phenotype common to multiple *sina* mutants and the formation of these polycomplexes is correlated with defects in chromosome segregation.

### The structure of *sina*-induced polycomplexes

**Fig 4:**
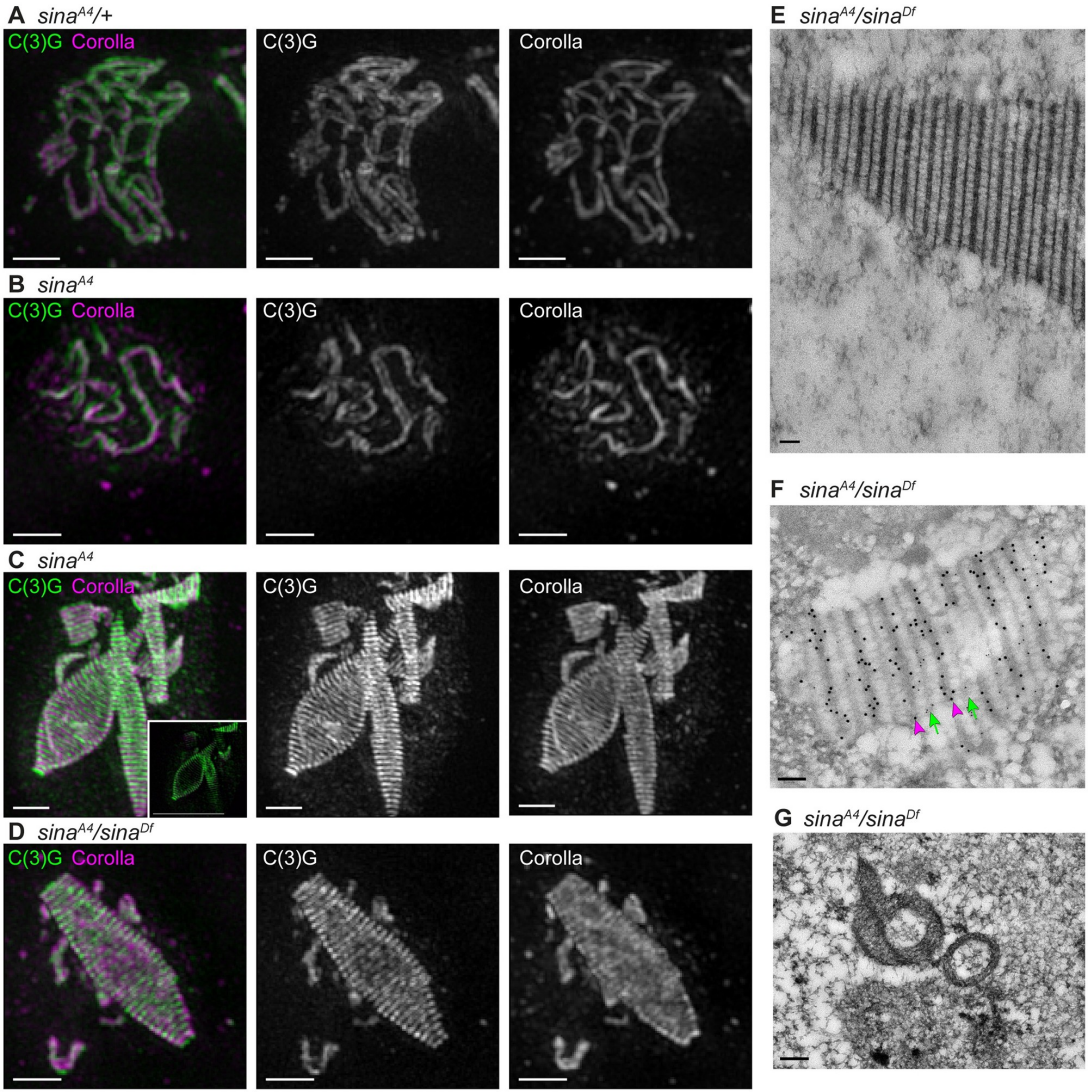
SIM and immuno-EM reveal a structure comprised of a repeating pattern of central region proteins. (A-D) SIM using an antibody recognizing the C-terminus of C(3)G (green), which localizes at the lateral element and is resolved as two tracks in wild type [8], and the central element component Corolla (magenta). Scale bars, 1 μm. Images are projections of nuclei from a larger *z*-stack. (A) *sina*^*A4*^*/+* nucleus with wild-type tripartite SC. (B-C) *sina*^*A4*^ nuclei showing (B) a small region of SC in early pachytene with tripartite structure and (C) polycomplexes from a mid-prophase oocyte. Inset in (C) shows a single *z*-slice, illustrating that the central region components appear to be absent from the center of the large polycomplex. (D) *sina*^*A4*^*/sina*^*Df*^ nucleus with a large polycomplex. (E) EM image of a *sina*^*A4*^*/sina*^*Df*^ polycomplex, illustrating its repeating structure. Scale bars, 100 nm for (E-F). (F) Immuno-EM with the C-terminus of C(3)G labeled with small gold particles (green arrows) and Corolla labeled with larger gold particles (magenta arrowheads) on a polycomplex from a *sina*^*A4*^*/sina*^*Df*^ ovary. The C-terminus of C(3)G localizes to bands having similarities to wild-type lateral element lines in EM and alternates with Corolla, which localizes to bands having similarities to central element lines in wild type. (G) EM section through two polycomplexes in *sina*^*A4*^*/sina*^*Df*^ ovaries. The section is interpreted to be an oblique section through the polycomplex on the left based on a similar-looking EM section of a polycomplex described in [45]. The section illustrates the polycomplexes have a hollow inside appearance. Scale bar, 0.2 μm.

We used structured illumination microscopy (SIM) to examine the organization of the abnormal SC structures in greater detail. Using SIM, the LEs of a normal SC can be resolved as two parallel tracks along the chromosome arms with an antibody recognizing the C-terminus of C(3)G. Similarly, the CE can be identified with an antibody recognizing Corolla, which localizes between these two parallel tracks [8,17]. This wild-type, tripartite SC pattern was observed in *sina*^*A4*^*/+* heterozygotes, as expected given that the *sina*^*A4*^ mutation is recessive (Fig 4A). Examination of *sina*^*A4*^ homozygotes by SIM revealed stretches of tripartite SC in early pachytene (region 2A) (Fig 4B), indicating that *sina*^*A4*^ can assemble some tracks of visually normal-looking SC.

We next examined the polycomplexes in both *sina*^*A4*^ homozygotes and *sina*^*A4*^/*sina*^*Df*^ females using the C-terminal C(3)G and Corolla antibodies. SIM revealed alternating layers of Corolla and C(3)G C-terminus along the length of the polycomplex structures (Fig 4C and 4D). In both genotypes the C(3)G and Corolla proteins appeared to localize only on the outside surface of the larger structures (inset, Fig 3C). Morphologically, the structures looked like rods, cones, or complex shapes (Fig 4C and 4D).

The presence of repeated alternating layers of SC proteins has been shown to be a characteristic of polycomplexes [27]. This repeating pattern supports our earlier assessment that the aberrant SC structures in *sina* mutants are polycomplexes. Polycomplexes have been observed in Drosophila oocytes as well as in other organisms [12,22,23,24,26,28,45,46,47]. However, the polycomplexes found in *sina* mutants are more numerous and tend to be longer than more recently characterized examples in Drosophila [12,45].

To further characterize the architecture of the polycomplexes, *sina*^*A4*^*/sina*^*Df*^ ovaries were examined using electron microscopy (EM). EM revealed a clear alternating pattern of electron-dense lines that resembled the LE and central element lines observed by EM of wild-type SC (Fig 4E and 4F) [12]. Immuno-EM with antibodies to Corolla and the C-terminus of C(3)G revealed that Corolla, which localized to the denser lines resembling the CE in wild-type SC, alternated out-of-phase with the C-terminus of C(3)G, which localized to the less-dense lines resembling LEs in wild-type SC (Fig 4F). A similar repeating pattern has been previously observed by EM in Drosophila [12]. An EM image of what appears to be a slice through two polycomplexes supports what was observed by SIM for the larger polycomplexes, that the larger polycomplexes have the appearance of a hollow middle (Fig 4G). Similar views of previously characterized polycomplexes observed by EM in mutants that only express a version of C(3)G lacking the C-terminus also appeared to be hollow in the middle [45].

Since the central region proteins Corolla, C(3)G, and Cona are mutually dependent on each other for wild-type SC formation [7,8], we next examined whether Cona was present in the *sina* polycomplexes. Both an antibody recognizing Cona and a Venus-tagged Cona overexpression construct localized to the polycomplexes in *sina*^*A4*^ and *sina*^*A4*^*/sina*^*Df*^ ovaries (S5 Fig). By SIM, the Venus-tagged Cona incorporated robustly into the large polycomplexes in *sina*^*A4*^ ovaries and was located between the layers formed by the C-terminal C(3)G antibody (S5E Fig), similar to Corolla staining (Fig 4C). Taken together, these results support the conclusion that mutations in *sina* lead to the formation of numerous, large polycomplexes during prophase in Drosophila females. Moreover, the repeating array of SC proteins illustrates that the *sina* polycomplexes have an organized structure and are not merely amorphous aggregates of SC proteins.

### Cohesins and the lateral element protein C(2)M localize to a subset of *sina* polycomplexes

While polycomplexes have been observed previously in Drosophila oocytes and other organisms, their protein composition has been extensively examined in only a few organisms [21,22,25,29,30,31]. In a number of these investigations, the polycomplexes do not appear to be associated with DNA [22,23,24,29]. Additionally, examples of polycomplexes lacking LE proteins have been identified. For example, in *Caenorhabditis elegans*, cohesin and axial/ LE proteins fail to localize to polycomplexes that result from a mutation in the LE protein *htp-3* [22,29]. Polycomplex-like structures have even been observed in mammalian cell lines when only the primary transverse filament protein was overexpressed [33].

**Fig 5:**
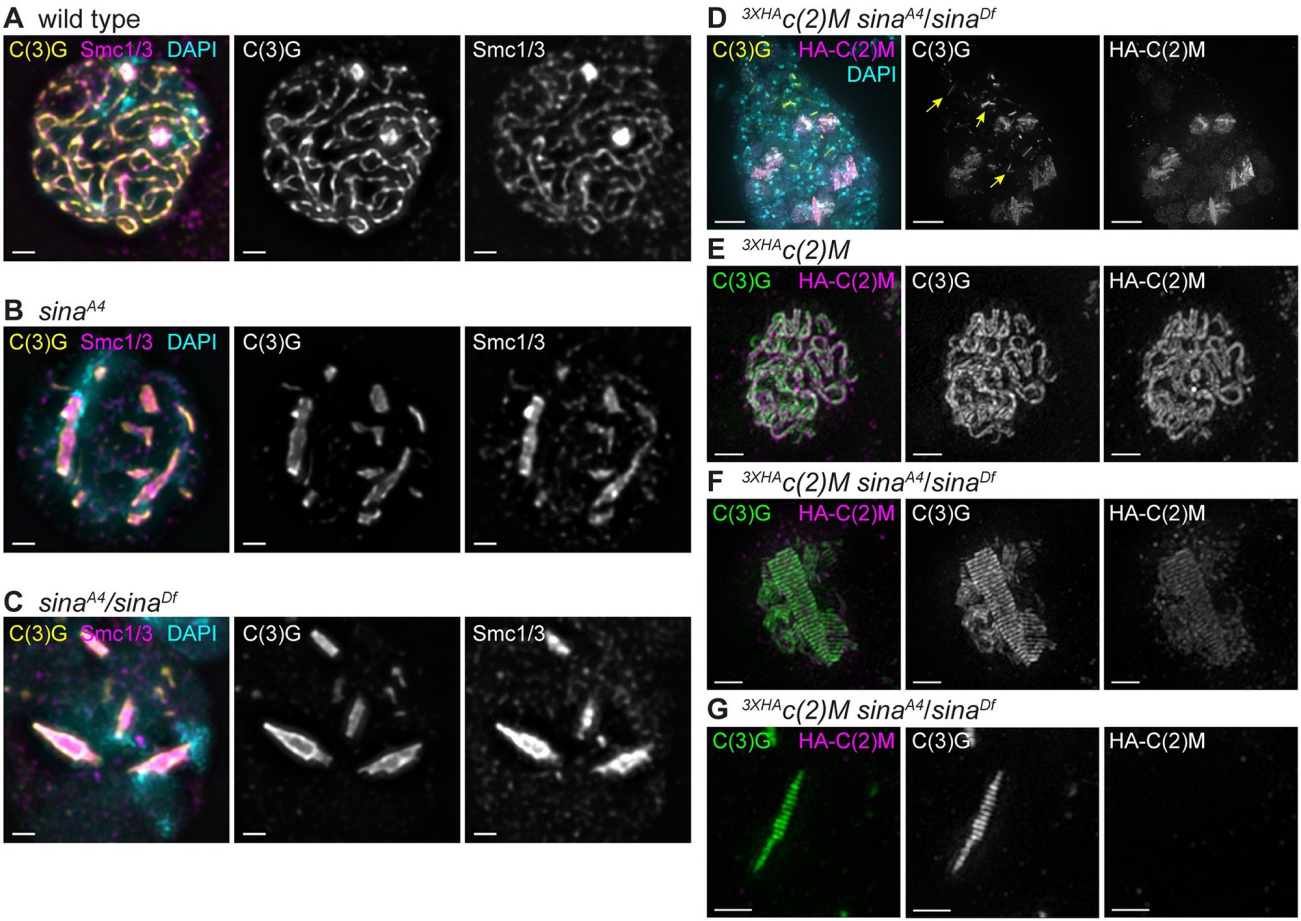
Lateral element proteins localize to the *sina* polycomplexes. (A-C) Using a chromosome spread protocol, Smc1/3 antibodies (magenta) localize to the *sina* polycomplexes marked with C(3)G (yellow). DAPI is labeled in cyan. Scale bars, 1 μm. (A) Wild-type nucleus with Smc1/3 localizing to the chromosome axes. (B) *sina*^*A4*^ and (C) *sina*^*A4*^*/sina*^*Df*^ nuclei with Smc1/3 localizing to the chromatin-bound polycomplexes. (D) Whole-mount image of premeiotic region 1 (top of image) and early pachytene (region 2A; bottom of image) from a *sina*^*A4*^*/sina*^*Df*^ germarium overexpressing ^*3XHA*^*c(2)M* using *nosGAL4*. The ^*3XHA*^*c(2)M* (recognized with an HA antibody in magenta) localizes to polycomplexes (labeled with C(3)G in yellow) in early pachytene. DAPI is in cyan. Yellow arrows indicate examples in the premeiotic region and early pachytene (region 2A) of polycomplexes containing C(3)G but lacking ^3XHA^C(2)M. Scale bar, 5 μm. (E-G) SIM images from *nosGAL4;* ^*3XHA*^*c(2)M* germaria. Scale bars, 1 μm. Images are projections from larger *z*-stacks. (E) A wild-type nucleus with the C-terminus of C(3)G (green) and ^*3XHA*^*c(2)M* (magenta) resolved as two tracks. (F-G) In *sina*^*A4*^*/sina*^*Df*^ nuclei, the ^*3XHA*^*c(2)M* protein shows two localization patterns: (F) shows a repeated pattern of ^*3XHA*^*c(2)M* similar to the C-terminus of C(3)G while (G) shows a polycomplex devoid of ^*3XHA*^*c(2)M*.

To examine the polycomplexes in *sina*^*A4*^ and *sina*^*A4*^*/sina*^*Df*^ mutants for the association of chromatin and LE proteins, we used a spread protocol in which soluble proteins are removed and only those proteins that are chromatin-associated remain bound to the slide. As was observed for the SC in wild-type nuclei (Fig 5A), we found that polycomplexes could be readily identified within nuclei in chromosome spreads of *sina*^*A4*^ and *sina*^*A4*^*/sina*^*Df*^ ovaries (Fig 5B and 5C). While this protocol would fail to reveal the presence of a subpopulation of non-chromatin-associated polycomplexes, these studies provide evidence that at least some of the *sina* polycomplexes are attached to chromatin. The polycomplexes in Fig 5B and 5C are entirely within the DAPI-stained regions suggesting that the polycomplexes in these nuclei are attached to the chromatin at multiple points along the polycomplexes.

As described in Methods, we used a cocktail of antibodies to both Structural maintenance of chromosomes 1 and 3 (Smc1/3) to examine the localization of these core cohesin components to the *sina* polycomplexes (Fig 5B and 5C). In wild type, Smc1/3 can be observed as tracks similar to the C-terminus of C(3)G (Fig 5A) as has been reported previously [48]. In chromosome spreads of *sina*^*A4*^ and *sina*^*A4*^*/sina*^*Df*^ mutants, the Smc1/3 antibodies localized to the *sina*-induced polycomplexes (Fig 5B and 5C). To the best of our knowledge, the presence of core cohesin proteins has not been reported for polycomplexes previously characterized in Drosophila, although a recent example of polycomplexes in *C*. *elegans* does appear to contain SMC3 [31].

We also examined the kleisin-like LE protein C(2)M (Crossover Suppressor on 2 of Manheim) with respect to *sina*-induced polycomplexes by using an N-terminal HA-tagged C(2)M overexpression construct (^3XHA^c(2)M) [9]. In whole-mount preparations this construct localized to many, but not all, of the SC polycomplexes in *sina*^*A4*^*/sina*^*Df*^ oocytes (Fig 5D). By SIM, ^3XHA^C(2)M clearly localized in two tracks near the C-terminus of C(3)G in wild-type nuclei (Fig 5E) and in a striped pattern near the C-terminus of C(3)G for *sina*^*A4*^*/sina*^*Df*^ polycomplexes (Fig 5F). SIM also showed a failure of ^3XHA^C(2)M to localize to a subset of *sina*-induced polycomplexes (Fig 5G). Upon further examination of images of *sina*^*A4*^*/sina*^*Df*^ germaria, we found that the thin polycomplexes formed in the mitotic nuclei in region 1 consistently appeared to lack ^3XHA^C(2)M (Fig 5D). This was not surprising, because C(2)M is not normally expressed in region 1 of the germarium [9]. The few polycomplexes lacking ^3XHA^C(2)M in early pachytene (region 2A) were of a similar size to the polycomplexes formed in region 1. We speculate that the polycomplexes formed in the absence of C(2)M in region 1 may be unable to later recruit C(2)M as the nuclei enter meiosis in early pachytene (region 2A). Our current studies cannot differentiate whether the polycomplexes that form in the premeiotic divisions are a different structure that prevents the localization of C(2)M or whether C(2)M can only load when polycomplexes are formed de novo. Answering this question will require further investigation.

### Polycomplexes in *sina* mutants persist into late prophase and early prometaphase I

In wild-type Drosophila ovaries, up to four nuclei of the 16-cell interconnected cyst initiate SC assembly along the chromosome arms in early pachytene (region 2A). By mid-pachytene (region 3) all but one of the nuclei disassemble their SC (Fig 1B). The single remaining SC-positive nucleus becomes the oocyte, while the other formerly SC-positive nuclei assume a nurse cell fate. In *sina*^*A4*^/*sina*^*Df*^ germaria, all the nuclei that normally initiate SC assembly appear to form polycomplexes, while in *sina*^*A4*^ homozygotes, polycomplexes can be observed in both nuclei in the early/mid- pachytene (region 2B) cysts that still contain SC proteins (S2B and S2C Fig). Once formed, polycomplexes remained in mid-pachytene (region 3) in a subset of *sina* mutant nuclei that would have normally disassembled their SC to become nurse cells.

To determine if the persistence of nuclei with *sina*-induced polycomplexes was the result of a failure to specify the oocyte, we examined Orb localization in *sina* mutants. Orb is a protein required for the determination of the oocyte nucleus and concentrates in the cytoplasm of the specified oocyte [49,50]. In wild-type, as well as *sina*^*A4*^ and *sina*^*A4*^/*sina*^*Df*^ mutant ovaries, there was only a single nucleus accumulating Orb protein in mid-pachytene (region 3) (S2A–S2C Fig). This indicates that oocyte specification occurred normally and that the persistence of polycomplexes in the additional nuclei is likely caused by a disassembly delay (S2B and S2C Fig).

**Fig 6:**
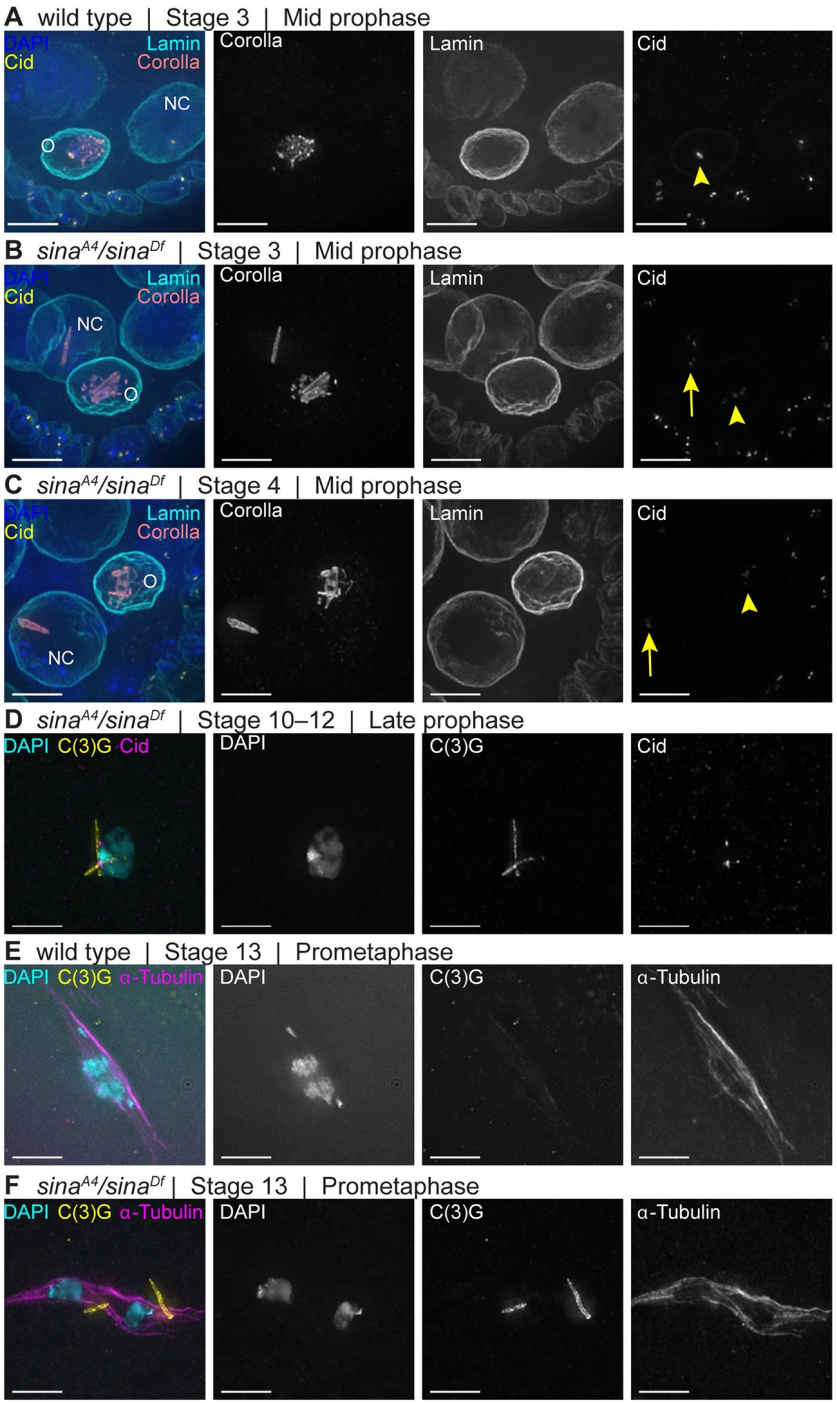
A subset of *sina* polycomplexes exhibit delayed disassembly. (A) A wild-type mid-prophase (stage 4) egg chamber. Centromeres are associated with the SC in the oocyte (yellow arrowhead) nuclei. (B-C) *sina*^*A4*^*/sina*^*Df*^ egg chambers at mid-prophase stage 3 (B) and stage 4 (C) display polycomplexes in nurse cells (NC) and oocyte (O) nuclei. Centromeres are associated with the polycomplexes in both nurse cell (yellow arrows) and oocyte (yellow arrowheads) nuclei. DAPI (blue), Corolla (coral), Lamin (cyan) and Cid (yellow). (D) A late prophase *sina*^*A4*^*/sina*^*Df*^ oocyte that continues to show the presence of polycomplexes. The polycomplexes (labeled with C(3)G antibody in yellow) appear to have regions that associate with DNA (cyan), including centromeres (magenta). (E) Wild-type prometaphase I oocyte lacking any structures that contain C(3)G (yellow). (F) *sina*^*A4*^*/sina*^*Df*^ prometaphase I oocyte with small C(3)G-positive structures associated with the spindle (α-Tubulin in magenta). DAPI (cyan) shows chromosomes that are not maintained at the middle of the spindle. Scale bars, 5 μm. Images are partial projections of *Z*-stacks.

SC is normally present only in the oocyte nucleus in mid-prophase in wild type (Fig 6A). Polycomplexes were observed to persist in nurse cells in mid-prophase (stages 2–9) in *sina*^*A4*^/*sina*^*Df*^ oocytes, including in nurse cell nuclei that had begun their endoreduplication cycles as based on DAPI (Fig 6B and 6C). There were typically only one or two persisting polycomplexes per nurse cell nucleus, while multiple polycomplexes could be observed in the four nuclei building polycomplexes in early pachytene (Table 2). This demonstrates that only a subset of the polycomplexes resisted timely disassembly in the nurse cell nuclei of *sina* mutants.

More intriguingly, a subset of the polycomplexes within *sina*^*A4*^/*sina*^*Df*^ oocyte nuclei were also resistant to disassembly. While SC progressively disassembles along the chromosome arms in stages 5–7 in wild type, polycomplexes could still be observed in *sina*^*A4*^/*sina*^*Df*^ oocytes that appeared to be in late prophase (stages 10–12). Later-stage polycomplexes may not be associated along their entire lengths with the chromatin, since examples could be identified that appeared to associate at one end in the chromatin while the other end of the polycomplex extended past the DAPI staining (Fig 6D).

Most surprisingly, while central region components are absent during prometaphase I in wild-type oocytes (Fig 6E), polycomplex-like structures could be observed after spindle assembly in *sina*^*A4*^/*sina*^*Df*^ oocytes (Fig 6F). These polycomplexes were still present in 81.6% of *sina*^*A4*^/*sina*^*Df*^ prometaphase I/ metaphase I oocytes (n = 98). They were either associated with the meiosis I spindle or pushed free into the cytoplasm rather than near the chromosomes. In Drosophila females, the chromosomes promote meiosis I spindle assembly, so it is possible the growing microtubules dislodge any remaining polycomplexes near the chromosomes. Polycomplexes were not observed after spindle assembly in *sina*^*A4*^ oocytes (n = 46), and no SC structures were observed in wild-type after spindle assembly (n = 37) (Fig 6E). The ability of the polycomplexes to persist after spindle assembly in *sina*^*A4*^/*sina*^*Df*^ demonstrates that at least a subset of the polycomplexes resist timely disassembly. While polycomplexes have been reported after spindle assembly in other organisms, this is the first observation of polycomplexes present during prometaphase I of Drosophila oocytes [23,27].

### Centromeres associate near *sina* polycomplexes and centromere clustering is defective in *sina* mutants

The central region components of the SC are first loaded at the centromeres in the pre-meiotic dividing nuclei, and the centromeres are the last place the SC components are disassembled at mid/late prophase [10,11,41]. We examined the localization of centromeres with respect to the polycomplexes in comparison to wild-type nuclei (S6A Fig). We used an antibody recognizing the centromere-specific histone Centromere identifier (Cid) [51] to mark centromeres, a Corolla antibody to identify polycomplexes, and an antibody against lamin to clearly demarcate nuclear bounds and assist with scoring individual nuclei (S6 Fig). The use of the lamin antibody demonstrated the polycomplexes were confined to the nucleus (S6 Fig). Small polycomplexes were observed in the pre-meiotic nuclei in region 1 that were associated with one or more centromeres in *sina*^*A4*^*/sina*^*Df*^ nuclei, similar to the foci of SC components associated with centromeres in region 1 of wild type (S6B and S6C Fig) [41]. In early pachytene (region 2A) and throughout early/mid-prophase, centromeres appeared associated near one or more of the polycomplexes in *sina*^*A4*^*/sina*^*Df*^ ovaries (S6D and S6E Fig). While only a subset of the *sina* polycomplexes were near centromeres, nearly all centromeres were observed near a polycomplex in pachytene. In the hypomorphic *sina*^*A4*^ mutant, we observed Cid foci associated with the tracks of SC in early pachytene (region 2A) in a manner similar to wild type (S6A Fig), but centromeres associated with the polycomplexes once polycomplex formation was initiated (S6F and S6G Fig). Examples where the centromeres appeared to be organized along or around the polycomplexes in *sina* mutants were observed (S6 Fig). This close association of the centromeres to polycomplexes is more apparent when examining centromeres using stimulated emission depletion (STED) microscopy (S7 Fig). In the nurse cells that maintained polycomplexes into mid-prophase, the remaining polycomplexes were observed to also remain associated with Cid foci (Fig 6B and 6C).

**Fig 7:**
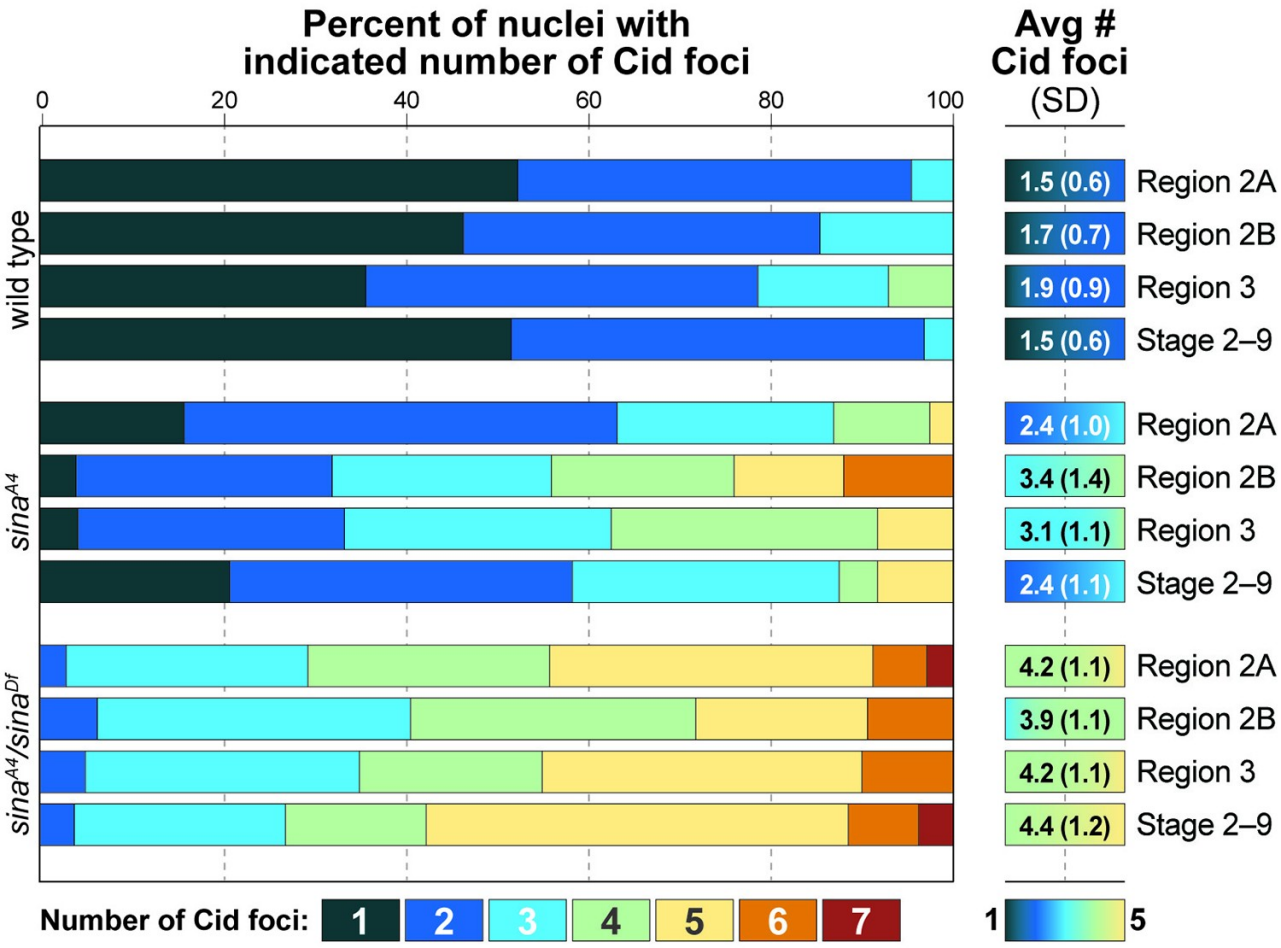
Centromere clustering is abnormal in *sina*^*A4*^*/sina*^*Df*^ mutants. The percentage of nuclei showing the indicated number of centromere clusters is displayed by color with centromere cluster average and standard deviation on the right. Number of nuclei scored for wild type: 65 (region 2A), 41 (region 2B), 14 (region 3), and 31 (stages 2–9); *sina*^*A4*^: 38 (region 2A), 25 (2B), 24 (Region 3), and 24 (Stages 2–9); *sina*^*A4*^*/sina*^*Df*^ 34 (region 2A), 32 (region 2B), 20 (region 3), and 26 (stages 2–9). For both *sina*^*A4*^ and *sina*^*A4*^*/sina*^*Df*^ nuclei, centromere clustering was significantly different at all stages from wild type of the same stage at *p* values of <0.05 (statistical test, two-tailed Mann-Whitney test).

The SC has been shown to be required for the process of centromere clustering in Drosophila oocytes [10,11]. In wild-type Drosophila oocytes, the centromeres from the four sets of chromosomes form an average of approximately two clusters (Fig 7 and S6A Fig) [10,11]. We examined whether the process of centromere clustering was affected in *sina* mutant ovaries. While centromeres can associate with the *sina* polycomplexes, *sina* mutant ovaries failed to promote and maintain centromere clustering. Consistent with previously published results [10,11], in wild-type nuclei an average of 1.5 Cid foci were observed in early pachytene (region 2A) and a similar level of clustering was maintained into mid-prophase (Fig 7). In early pachytene (region 2A) in *sina*^*A4*^ homozygotes, where some nuclei have assembled full or partial SC tracks, there was an average of 2.4 Cid foci (Fig 7). However, as the cyst progresses through pachytene, the increase in polycomplex formation was correlated with a worsening of centromere clustering, averaging 3.4 Cid foci at early/mid-pachytene (region 2B). Subsequently, centromere clustering improved at mid-prophase to 2.4 Cid foci. The analysis of centromere cluster number in *sina* mutants was complicated by the varying patterns of Cid localization observed. In some oocytes, Cid appeared as elongated smears wrapping along or around the polycomplexes, thus requiring the nuclei with the more aberrant Cid localization patterns to be excluded from quantification (S6E Fig).

In *sina*^*A4*^*/sina*^*Df*^ ovaries centromere clustering was more severely disrupted. In early pachytene (region 2A), *sina*^*A4*^*/sina*^*Df*^ nuclei had an average of 4.2 Cid foci, with the number of Cid foci varying from one to seven (Fig 7 and S6D Fig). Centromere clustering remained defective in *sina*^*A4*^*/sina*^*Df*^ ovaries throughout pachytene (Fig 7). An average of four Cid foci usually indicates that pairing of homologous centromeres is occurring but clustering of non-homologous chromosomes is disrupted (as Drosophila has four pairs of homologous chromosomes). While the average number was four Cid foci, there were examples of nuclei with five or more Cid foci in *sina*^*A4*^*/sina*^*Df*^ ovaries, indicating that in some nuclei the pairing of homologous centromeres can also be disrupted in *sina*^*A4*^*/sina*^*Df*^ ovaries (Fig 7). As we showed above, centromeres are able to associate near the *sina*-induced polycomplexes, but disruption of *sina* prevents centromere clustering when polycomplexes form. We cannot rule out that the process of centromere clustering is directly affected by the absence of *sina* function. However, we prefer a model in which that it is the presence of the polycomplexes that disrupts the ability of the centromeres to cluster. The weaker defect in centromere clustering observed in early pachytene (region 2A) in *sina*^*A4*^ homozygotes (before full polycomplex formation) further supports the hypothesis that polycomplexes disrupt centromere clustering and, again, that *sina*^*A4*^ is a hypomorphic mutation.

The centromeres or centromere-associated proteins may have an affinity for some feature of the polycomplexes, and/or centromeres may act as an initiation point for polycomplex formation. The polycomplexes that persisted into later stages in both the pro-oocyte and nurse cells frequently were associated with centromeres (Fig 6B and 6C). Potentially, the centromeres are involved in the mechanism preventing the disassembly of a subset of polycomplexes, similar to the delayed disassembly of SC components near the centromeres in wild type.

### The *sina*^*A4*^ mutation causes decreased recombination

Null mutations in central region components of the SC cause a reduction in the number of meiotic DSBs, abolish crossover formation, and subsequently cause high levels of chromosome nondisjunction [6,7,8]. This is likely due to the failure of central region mutants to assemble SC. In *sina* mutant germaria, however, SC components primarily assemble into aberrant polycomplexes. We wanted to determine if the *sina*-induced SC polycomplexes were able to function in the formation of crossovers, and therefore we analyzed both the presence of meiotically induced DSBs and the repair of those DSBs into crossover products in *sina* mutant females.

**Fig 8:**
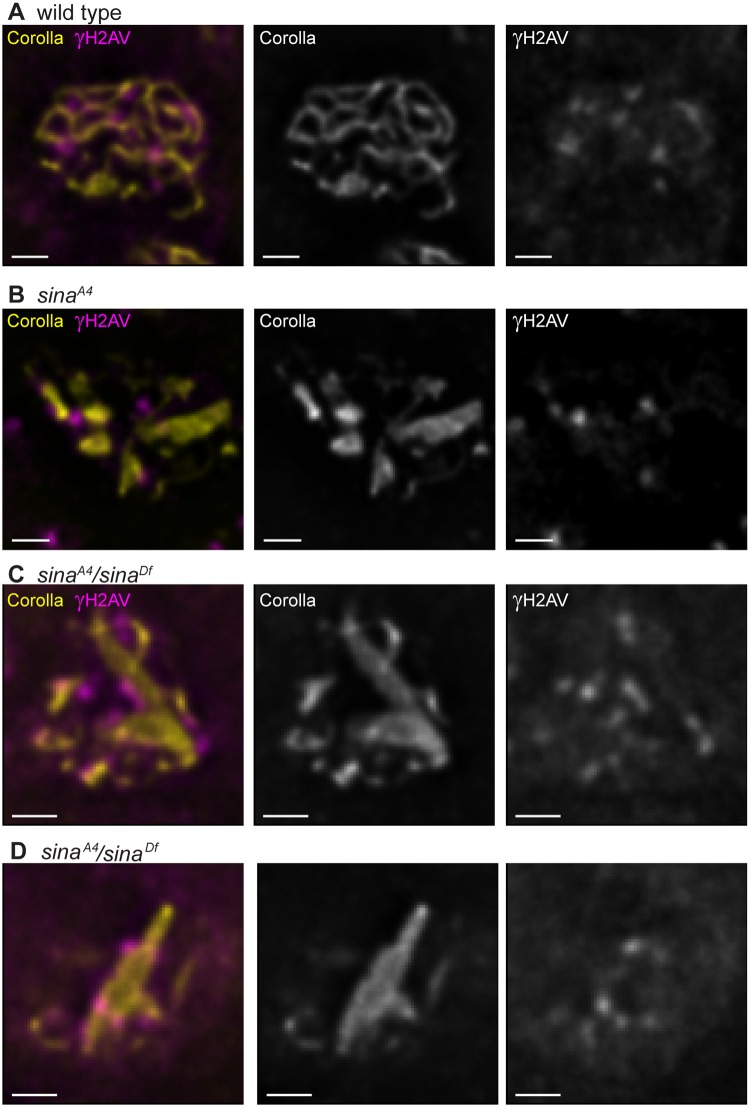
DSBs are located near polycomplexes in *sina* mutants. Sites of DSBs were labeled with a γH2AV antibody (magenta). SC is labeled with Corolla (yellow). (A) In a wild-type nucleus, sites of DSBs associate with the SC. (B) In a *sina*^*A4*^ nucleus, γH2AV foci are associated with SC tracks and polycomplexes. (C-D) In the *sina*^*A4*^*/sina*^*Df*^ nuclei, the sites of DSBs associate with the polycomplexes. Scale bar, 1 μm. Images are a single slice from larger *z*-stacks for clarity of the γH2AV localization.

We identified DSBs using an antibody that recognizes γH2AV, the histone modification made in response to DSB formation. In wild-type oocytes, γH2AV foci are first observed in early pachytene (region 2A) of the germarium, following the formation of full-length SC (Fig 8A) [52]. Foci persist into early/mid-pachytene (region 2B), and steadily decrease in number as cysts progress into mid-pachytene. Very few γH2AV foci are observed in mid-pachytene (region 3), indicating repair of DSBs into crossovers and noncrossovers has been initiated. In both *sina*^*A4*^ and *sina*^*A4*^/*sina*^*Df*^ ovaries, γH2AV foci could be observed at a similar frequency as wild type in early pachytene (Fig 8B–8D, Table 4), demonstrating that the induction of meiotic DSBs is not disrupted in *sina* mutants. In addition, the kinetics of DSB repair in *sina* mutants was not significantly different than wild-type (Table 4). Taken together these results suggest that DSB formation and repair are not significantly disrupted in the absence of Sina function.

**Table 4:** Double-strand break formation and repair.

Genotype	Avg # γH2AV foci	SD^b^	Nuclei scored
**Early pachytene (region 2A)**^c^			
wild type^a^	10.5	2.3	13
*sina*^*A4*^	12.2	3.5	12
*sina*^*A4*^*/sina*^*Df*^	11.9	3.6	14
**Mid-pachytene (region 3)**^c^			
wild type	0	0	10
*sina*^*A4*^	0.1	0.3	13
*sina*^*A4*^*/sina*^*Df*^	0.6	0.8	13

^*a*^ Wild type = *y w; spa*^*pol*^.

^*b*^ Standard deviation

^c^ Neither mutant is statistically significant from wild type at either early or mid-pachytene (statistical test, two-tailed Mann-Whitney test, all p values >0.05).

In our analysis, we observed that many of the γH2AV foci appeared to be in close proximity to polycomplexes when present in *sina* mutants (Fig 8B–8D). Therefore, we analyzed the position of the γH2AV foci in relation to the SC for both wild-type and *sina* mutants. We found in wild-type the γH2AV foci were associated with tracks of SC 100% of the time in both early pachytene (52 γH2AV scored from 5 nuclei) (Fig 8A) and early/ mid-pachytene nuclei (28 γH2AV scored from 10 nuclei). The vast majority of γH2AV foci were also found associated with SC in both *sina*^*A4*^ and *sina*^*A4*^*/sina*^*Df*^ from early pachytene throughout early/mid-pachytene. In *sina*^*A4*^ we found that 100% of the γH2AV foci were associated with SC in early pachytene nuclei containing polycomplexes (92.8% associated with polycomplexes, 7.2% associated with track-like SC, 70 γH2AV scored from 6 nuclei). Similar association was observed in early/mid-pachytene (85.3% associated with polycomplexes, 14.7% associated with track-like SC, 34 γH2AV foci scored from 10 *sina*^*A4*^ nuclei). In early pachytene *sina*^*A4*^*/sina*^*Df*^ nuclei the γH2AV foci were 89.0% associated with polycomplexes and 9.6% associated with tracks or foci of SC (73 γH2AV foci scored from 7 nuclei). In early/mid-pachytene *sina*^*A4*^*/sina*^*Df*^ nuclei the γH2AV foci were 90.0% associated with polycomplexes and 3.3% percent associated with a focus of SC (30 γH2AV foci scored from 14 nuclei).

Examples of the γH2AV signal appearing to form patches along or around the polycomplexes could be observed in some *sina* mutant nuclei, making it difficult to assign a location and number of γH2AV foci in these nuclei. These patches of signal may represent association of multiple closely spaced DSBs, but we could not rule out the localization pattern representing a spread of the γH2AV signal from one DSB. While sites of DSBs localize near *sina* polycomplexes the DSBs may not be interacting directly with the central region components of the *sina* polyocomplex. Proteins, such as the DSB initiation and repair machinery could be mediating the localization pattern of γH2AV foci in regions near the polyocomplexes.

Since DSBs occur at wild type frequency in *sina* mutant oocytes and can associate with the SC tracks and polycomplexes, we next asked if the DSBs could be converted to crossovers in these mutants. Meiotic recombination along the entire length of the *X* chromosome was assayed in *sina*^*A4*^ and *sina*^*A4*^/*sina*^*Df*^ females and compared to wild type. For *sina*^*A4*^ homozygotes, the total recombination along the *X* chromosome was reduced (to ~20% of wild type), particularly for the *sc–cv* and *cv–v* intervals, which are the intervals most distal to the centromere (Table 5). In *sina*^*A4*^/*sina*^*Df*^ females, recombination was further reduced (to ~11% of wild type) but was not abolished. Although DSBs can be recruited to the *sina* polycomplexes, the polycomplexes may not allow for the efficient maturation of DSBs into crossovers between homologous chromosomes. The failure to form crossovers likely contributes to the high *X* chromosome nondisjunction of *sina*^*A4*^/*sina*^*Df*^ females. The higher level of recombination in *sina*^*A4*^ homozygotes correlates with both the lower levels of *X* chromosome nondisjunction and the increase in track-like SC observed in early pachytene (region 2A) in this mutant background.

**Table 5:** *X* chromosome recombination.

	Frequency of crossing over (%)^a^					
Genotype^b^	*sc–cv*	*cv–v*	*v–f*	*f–y*^+^	Total	*n*^c^
wild type	10	19.3	21.6	12.2	63.1	1197
*sina*^*A4*^	0.6 (6.0)	0.9 (4.6)	6.1 (28.2)	5.2 (42.6)	12.8 (20.3)	326
*sina*^*A4*^*/sina*^*Df*^	0.6 (6.0)	3.0 (15.5)	1.2 (5.6)	2.4 (19.7)	7.2 (11.4)	165

^*a*^ Parentheses = percent of wild-type crossing over

^*b*^ Wild type = *y sc cv v f y+/y w*. *sina*^*A4*^ = *y sc cv f y+/y; sina*^*A4*^. *sina*^*A4*^*/sina*^*Df*^ = *y sc cv v f y+/y w; sina*^*A4*^*/sina*^*Df*^.

^*c*^ Only accurately segregating female progeny were scored.

Our observation that crossover formation is decreased in *sina*^*A4*^*/sina*^*Df*^ females is consistent with the analysis of prometaphase I/metaphase I oocytes. Chiasmata are required to hold homologous chromosomes at the metaphase I plate. If oocytes fail to make a crossover on at least one pair of homologs, the chromosomes will not remain at the metaphase I plate. The chromosomes will instead move extremely far apart, in some cases nucleating multiple spindles, or will appear to enter anaphase I [53,54]. When examining prometaphase I/metaphase I *sina*^*A4*^*/sina*^*Df*^ oocytes, 69.4% (N = 98) of oocytes showed recombination-defective chromosome configurations (Fig 6F) that were absent in control oocytes (N = 37) (Fig 6E). Only 28.3% (N = 46) of *sina*^*A4*^ homozygotes displayed recombination-defective chromosome configurations, consistent with the lower chromosome nondisjunction observed for this mutant background. Track-like SC is also more prevalent in early pachytene in *sina*^*A4*^ germaria when DSBs are induced. It is possible that the DSBs that are matured into crossovers are those associated with track-like SC in *sina*^*A4*^ and the partial tracks in *sina*^*A4*^/ *sina*^*Df*^. Taken together these data suggest that the *sina* polycomplexes cause a defect with respect to crossover formation, despite the localization of DSBs near the polycomplexes.

### Loss of C(2)M leads to a reduction in polycomplex formation in *sina* mutants

**Fig 9:**
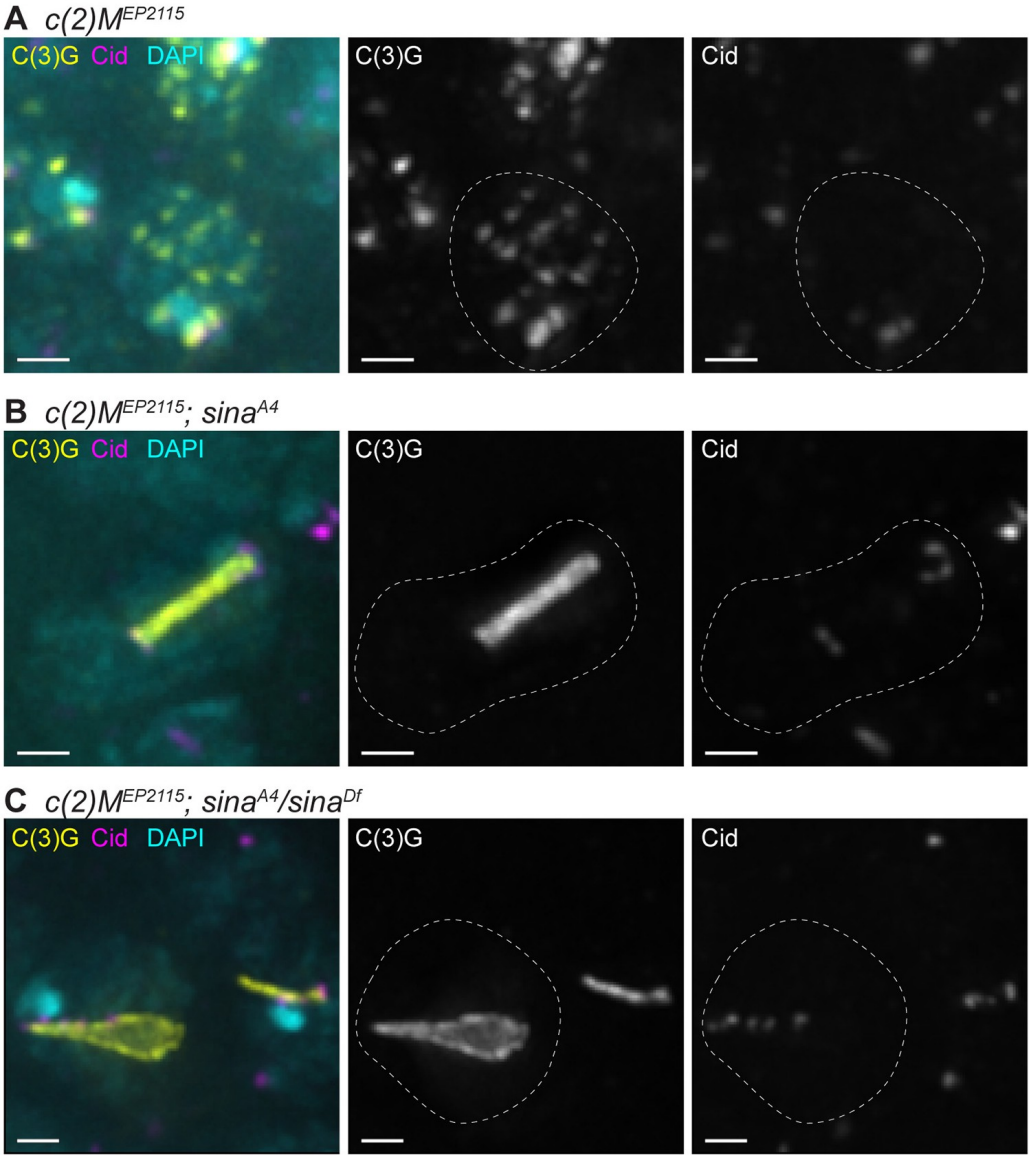
Loss of the LE protein C(2)M reduces but does not abolish polycomplex formation in *sina* mutants. Nuclei labeled with DAPI (cyan), C(3)G (yellow), and Cid (magenta). While *c(2)M*^*EP2115*^ (A) shows punctate SC staining, *c(2)M; sina* double mutants (B-C) display decreased polycomplex formation compared to *sina* mutants alone (see Fig 2). Scale bar, 1 μm. Images are projections from larger *z*-stacks.

Since the LE protein C(2)M localizes to many of the *sina* polycomplexes, we examined the consequences of loss of *c(2)M* on polycomplex formation. We generated females doubly homozygous for a null mutation in *c(2)M* (*c(2)M*^*EP2115*^) and *sina*^*A4*^, as well as *c(2)M*^*EP2115*^; *sina*^*A4*^*/sina*^*Df*^ females (Fig 9 and S8 Fig). In *c(2)M*^*EP2115*^ germaria, the SC begins to load as small patches near the centromeres and multiple additional locations, but fails to elongate the central region components into full tracks of SC (Fig 9A and S8D Fig) [9]. Surprisingly, in both double mutant backgrounds, the number of polycomplexes was reduced, but not eliminated, compared to each *sina* mutant alone (Table 2 and Fig 9B and 9C and S8 Fig, see Fig 2 for *sina* mutants alone). In *c(2)M*^*EP2115*^; *sina*^*A4*^*/sina*^*Df*^ nuclei, there was an average of 1.3 polycomplexes in early pachytene (region 2A) compared to 3.8 polycomplexes in *sina*^*A4*^*/sina*^*Df*^ alone (Fig 9C, Table 2). This difference in average polycomplex number was even greater in subsequent stages (Table 2). The phenotype in *c(2)M*^*EP2115*^; *sina*^*A4*^ females was more complex. In early pachytene (region 2A), *c(2)M*^*EP2115*^ mutants display numerous puncta of SC staining, while *sina*^*A4*^ mutants display mostly track-like SC in the earliest cysts and then form many thin polycomplexes. In *c(2)M*^*EP2115*^; *sina*^*A4*^ germaria, only puncta of SC could be observed in 6/37 early pachytene (region 2A) nuclei. Polycomplexes were present in the remaining early pachytene nuclei of *c(2)M*^*EP2115*^; *sina*^*A4*^ females with an average of 1.5 polycomplexes compared to 7.7 polycomplexes in *sina*^*A4*^ single mutants (Table 2). This difference in polycomplex number between the two genotypes continued in later stages (Table 2).

Intriguingly, the 1–2 polycomplexes in both *c(2)M*^*EP2115*^; *sina*^*A4*^ and *c(2)M*^*EP2115*^; *sina*^*A4*^*/sina*^*Df*^ nuclei were frequently associated near centromeres, with association defined as Cid signal touching the SC signal [41] (Fig 9B and 9C). At mid-pachytene in *c(2)M*^*EP2115*^; *sina*^*A4*^ (region 3) 90% of polycomplexes were associated with Cid (10 polycomplexes scored from 8 nuclei) and in *c(2)M*^*EP2115*^; *sina*^*A4*^*/sina*^*Df*^ nuclei 100% of polycomplexes (21 polycomplexes scored from 18 nuclei) were associated with Cid.

As with the single *sina* mutants, the polycomplexes in *c(2)M*^*EP2115*^; *sina*^*A4*^*/sina*^*Df*^ showed a large variation in length and width (Fig 3 and S3 Fig, Table 3), with less variability in polycomplex width in *c(2)M*^*EP2115*^; *sina*^*A4*^ oocytes in early pachytene. However, we did observe in *c(2)M*^*EP2115*^; *sina*^*A4*^ oocytes the polycomplex width became more variable at later stages (Fig 3 and S3 Fig, Table 3).

These results indicate that while C(2)M localizes to many of the polycomplexes in *sina* mutants, it is not required for the formation of all polycomplexes. The propensity for nuclei with 1–2 polycomplexes instead of multiple polycomplexes in *sina*^*A4*^ mutants indicates that C(2)M facilitates the initiation of polycomplexes, perhaps similar to how C(2)M is required for the elongation of central region components into long tracks along the chromosome arms in wild-type oocytes [9]. This idea is further supported by the observation that the 1–2 polycomplexes in each nucleus in the double mutants were often centromere associated. The loading of the central region components to the centromeres requires the Sunn, Solo and Ord protein complex in wild-type oocytes [10,48,55]. This same complex could be responsible for initiating polycomplexes near the centromeres and in the premeiotic region of the ovary, while initiation of additional polycomplexes at pachytene might be facilitated by a C(2)M-based protein complex. The different protein requirements of the polycomplexes in *sina* mutants indicates that polycomplex formation may not be uniform.

## Discussion

During meiosis, the components of the central region of the SC have the potential to misassemble into polycomplexes [23,27]. To prevent this process from impairing normal SC assembly, organisms likely have developed mechanisms to inhibit this form of SC assembly in meiosis. In Drosophila females, Sina appears to be a key regulator in preventing the polycomplex mode of assembly. Loss of Sina function allows SC components to preferentially incorporate into polycomplexes, leading to defects in the normal assembly of SC between homologous chromosomes.

### The function of Sina

Sina family members in multiple organisms have been shown to function as E3 ubiquitin ligases to target proteins for degradation. In the Drosophila eye, Sina, in combination with its cofactor Phyllopod, targets the transcriptional repressor Tramtrack for degradation to allow for R7 photoreceptor specification [36]. Closely related homologs of Sina in vertebrates have been implicated in the degradation of numerous proteins [34,35,37,38], supporting the idea that Sina might act during meiosis to mediate the degradation of a regulator of SC assembly in the oocyte. Indeed, we can imagine two variants on this hypothesis: 1) Sina-mediated ubiquitination functions primarily to stabilize those SC components that are properly assembled into SC between the homologs or 2) Sina-mediated ubiquitination acts to destabilize polycomplexes thus directing SC components to proper SC formation. That fact that in *sina*^*A4*^*/sina*^*Df*^ mutant oocytes we observed the formation of rod-like PC, even before canonical SC is formed supports the proposal that the primary role of wild-type Sina is to block PC formation.

Much of our ongoing studies focuses on identifying Sina targets, and specifically to determine whether Sina acts to induce the modification of SC structural components or rather regulators of SC assembly. If Sina directly degraded a SC component one would expect the phenotype of *sina* mutants to be nuclei with normal SC with additional polycomplexes due to the excess SC component self-assembling. *sina* mutant nuclei can be identified lacking normal SC and displaying only polycomplexes supporting the idea that Sina degrades a protein that regulates the choice between normal and polycomplex SC assembly.

Recent studies in other model systems have illustrated the roles of post-translational modifications, including phosphorylation and sumoylation, in controlling the assembly and disassembly of SC components along the chromosomes [5,18,19,20,21]. For example, expression in yeast of a version of the central region component Ecm11 that cannot be sumolyated results in the assembly of polycomplexes [21]. Knock-down of a proteasome component led to polycomplex formation in *C*.*elegans* [56]. In addition, ubiquitination has been associated with the regulation of meiotic recombination [56,57]. Mutation of mouse *siah1a*, a *sina* homolog, leads to defects at metaphase/ anaphase I in males [58]. While prophase appeared normal in the mutant male mice, this result suggests that ubiquitination, and specifically by Sina homologs, may play roles in regulating meiosis across species.

### How might the *A4* mutation affect Sina function?

The C-terminal half of Sina shows high sequence homology with its mammalian Siah1 and Siah2 homologs (see [16] for full description of the homology among the Sina family of proteins). This region of Sina homologs appears to mediate the binding of Sina to many of its targets and cofactors and to facilitate homo- and heterodimerization of Siah family members [59]. The *sina*^*A4*^ mutation described here resides in a C-terminal beta-sheet that mediates dimerization of human Siah1 in crystallography studies [59,60] and is absolutely conserved with the mammalian Siah1 and Siah2 proteins. The *sina*^*A4*^ mutation is an A to V amino acid change that behaves as a hypomorphic mutation. As an E3 ubiquitin ligase, Sina homologs interact with cell-specific cofactors to recognize targets for degradation [36,61,62]. The *sina*^*A4*^ mutation may impact the structure of Drosophila Sina and hamper its ability to recognize a specific meiotic cofactor/target(s). This hypothesis is supported by the observation that *sina*^*A4*^ mutant flies survive longer in our meiotic assays than null *sina* mutant flies, are partially fertile, and do not display obvious external mitotic defects [39,63]. This suggests the *A4* mutation disrupts Sina’s ability to target a protein(s) for degradation in the germline more severely than mitotic targets. Alternatively, the *sina*^*A4*^ mutation may be disrupting the stability of the Sina protein, particularly in the female germline.

### Sina acts at multiple points

Varying the dosage of wild-type Sina product clearly affects the onset of polycomplex formation. As noted above, in the hypomorphic *sina*^*A4*^ homozygotes, SC components attempt to assemble into track-like SC in early pachytene but then disassemble from the tracks and switch to polycomplex formation as the cyst progresses through the germarium. However, polycomplexes are seen earlier in *sina*^*A4*^*/sina*^*Df*^ mutant ovaries with small polycomplexes being observed in pre-mitotic nuclei in region 1 (at this stage in wild-type oocytes, central region components are normally only observed as foci associated with the centromeres).

We interpret these data to indicate that polycomplex assembly needs to be prevented (presumably by Sina) even before SC assembly, and likely at multiple stages in meiosis. Perhaps in homozygotes, the *sina*^*A4*^ mutant protein retains sufficient function until early pachytene to allow for SC assembly along the euchromatin, but ultimately fails to degrade enough of the target to prevent SC components from disassembling along the chromosome arms and initiating polycomplex formation. Moreover, the delayed appearance of polycomplexes in *sina*^*A4*^ homozygotes indicates that promotion of SC components into canonical SC may be an active process that needs to be continued throughout pachytene. Sina may be needed throughout pachytene to actively shift assembly of SC components away from self-assembling into polycomplexes.

### The persistence of *sina* polycomplexes beyond the end of prophase

It is unclear why in *sina*^*A4*^*/sina*^*Df*^ ovaries a subset of polycomplexes failed to be disassembled in late prophase and appeared to be maintained into prometaphase I (Fig 6). This persistence of polycomplexes until prometaphase I has been noted in other organisms [23,27]. In wild-type Drosophila females, the SC disassembles along the chromosome arms earlier than it does from the centromeres. This difference in the timing of SC disassembly indicates the oocyte must have a mechanism to differentiate between SC at different locations. A similar mechanism may mark some *sina*-induced polycomplexes as ready for disassembly in mid-prophase while others are marked to resist degradation at the time the SC is disassembled at the euchromatin in wild-type oocytes. The differential disassembly of *sina* polycomplexes may provide insight into the regulation of wild-type SC assembly and disassembly.

### The structure(s) of the polycomplexes

As is seen for the rare spontaneously occurring polycomplexes previously observed by EM in Drosophila [12,28], EM of the polycomplexes in *sina*^*A4*^*/sina*^*Df*^ mutants shows repeating layers of lateral and central elements. Both Corolla and Cona (the two known central element proteins in Drosophila) localize to the *sina* polycomplexes. Based on immuno-EM (Corolla) and SIM (Cona and Corolla), the proteins alternate with the C-terminus of C(3)G, which in wild type localizes to both LEs of the SC [8,12,17]. This arrangement suggests that the polycomplexes are composed of repeating layers of wild-type tripartite SC. How one “layer” of SC interacts with the next to form polycomplexes has been a question pondered for many years [23,27].

In *sina* mutants, the LE protein C(2)M localizes to most, but not all, of the polycomplexes in a pattern similar to the C-terminus of C(3)G (Fig 4F); this indicates that like in wild type, C(2)M localizes near the C-terminus of C(3)G. Since early *sina* polycomplexes lack C(2)M, this protein may not be required to connect the layers of the polycomplex but rather may play a role in initiating and promoting additional polycomplex formation. This idea is supported by the observation that polycomplex formation is reduced, but not abolished in *c(2)M; sina* double mutants (Fig 9 and S8 Fig). More intriguing is the localization of the core cohesin proteins Smc1/3 to *sina* polycomplexes. In *C*. *elegans*, SMC3 and meiotic kleisin proteins were recently observed to localize to polycomplexes in *akir-1; ima-2* double mutants, which likely have defects in the nuclear import and loading of cohesin proteins [31]. Conversely, core cohesin proteins in *C*. *elegans* were not observed to localize to polycomplexes caused by loss of meiotic kleisin proteins or the LE protein HTP-3 [22,25,29]. These examples, as well as the *sina* polycomplexes, illustrate that polycomplexes display variations between and within species despite exhibiting structural similarities.

### Why do *sina* polycomplexes have LE proteins?

Normally, the proteins C(2)M and the cohesins Smc1/3 are localized within the LE along the chromosome axes. In Drosophila, C(3)G spans the distance between the LEs, and by EM the C-terminus of C(3)G localizes at the LE [12]. One explanation for the localization of LE proteins to the polycomplexes is that the LE proteins are attracted to the C-terminal ends of C(3)G present in the *sina* polycomplexes. In the absence of competing central region components (as is the case for null mutants for central region components), the LE components remain associated with the chromosomes [7,9,48]. Alternatively, Sina may degrade a regulator that controls the localization of LE proteins in the homolog axes. Failure to degrade this regulator in *sina* mutants might drive the loss of LE proteins from the chromosome axes. Decreased LE proteins along the chromosome axis could prevent stable association of the central region along the homologs to promote polycomplex formation. Indeed, females mutant for both *c(2)M* and *ord*, which would disrupt both proposed meiotic cohesin complexes, display polycomplex formation indicating that loss of multiple lateral element protein complexes can cause polycomplex formation [11,55]. The possibility that Sina affects the localization of LE proteins would also be consistent with mechanisms of polycomplex formation in *C*. *elegans* in which loss of cohesin and LE proteins leads to polycomplex formation [22,25,29].

### Do the LE proteins mediate interaction with centromeres, DSBs and chromatin?

The presence of LE proteins could provide an explanation for the chromatin association of *sina* polycomplexes. While many previously studied polycomplexes do not show chromatin association [22,23,24], there is at least one reported example of DNA-associated polycomplexes. In a yeast mutant for the meiosis-specific transcription factor *ndt80*, DNA was shown in close association with the lines similar in appearance to the LEs of the polycomplexes as observed by EM [64]. Conversely, the polycomplexes in *ord; c(2)M* mutants, which would disrupt both proposed cohesin complexes, were seen in the cytoplasm indicating these polycomplexes are not chromatin associated [11,55]. Both observations are concordant with the idea that the LE proteins that associate with the *sina* polycomplexes may be mediating the binding of *sina* polycomplexes to chromatin.

While the *sina* polycomplexes are abnormal SC structures, there may well be aspects of their structure that resemble wild-type SC for both centromeres and DSBs (or centromere and DSB associated proteins) to localize near the polycomplexes. In wild-type oocytes, central region components first load to the centromeres and they are required for centromere clustering [8,10,41]. In yeast the transverse filament protein Zip1 also localizes early in meiosis to centromeres [65]. It is unclear how the SC interacts with the centromeres and how it is preferentially loaded to centromeres first in the mitotic divisions in wild-type flies. The centromeres or proteins that bind the centromeres may recognize some protein or substructure common to both wild-type SC and polycomplexes.

As is the case for centromeres, the recombination machinery may be able to recognize the polycomplexes as SC to affect the localization of DSBs. In Drosophila females the Vilya and Narya proteins, which are required for DSB formation and repair, initially localize to the central region of the SC, as well as to DSBs [66,67]. It is possible these and other DSB associated proteins are mediating the localization of DSBs near *sina* polycomplexes. However, the abnormal centromere clustering and decreased recombination in *sina* mutants indicates both processes require wild-type SC structure for completion. Decreased recombination is also associated with polycomplex formation in several *C*. *elegans* mutants [31,32]. The *sina* polycomplexes provide a tool for dissecting which aspects of SC structure are required for different SC functions.

### Model of Sina function in the germline

**Fig 10:**
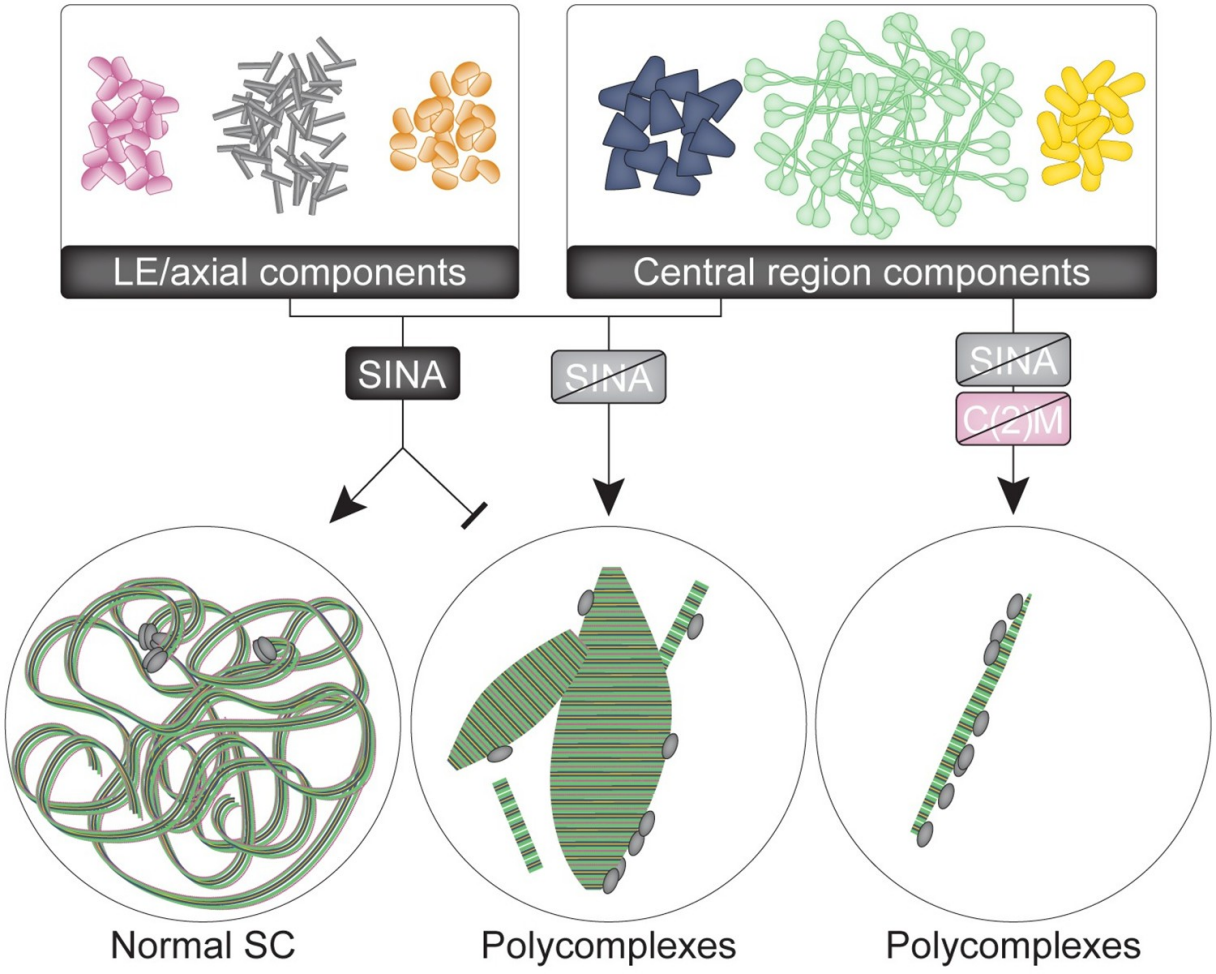
Model of Sina function in SC formation. The presence of functional Sina protein either promotes the assembly of SC components (both LE and central region components) into normal SC between homologous chromosomes and/or inhibits the assembly of SC components into multiple polycomplexes. Centromeres, which are clustered in wild-type nuclei, are shown as gray ovals. Loss of functional Sina protein shifts SC assembly into polycomplexes that interact with chromatin, including at centromeres. In the absence of both functional Sina and the LE protein C(2)M, fewer polycomplexes form, but the remaining polycomplexes associate with centromeres.

The observation of polycomplexes in numerous organisms illustrates that SC components have the intrinsic ability to polymerize into polycomplexes [13,22,23,24,25,27]. It would be beneficial to develop mechanisms to prevent polycomplex formation, which could interfere with the proper assembly of SC components to synapse the homologous chromosomes. Preventing polycomplex formation would also be crucial at times when SC components are expressed but chromosomes are not fully synapsed, such as during the final mitotic divisions of the 16-cell cyst in the Drosophila ovary when SC components are restricted to assembly only at the centromeres. The phenotypes of *sina* mutants in the germline suggest that Sina plays a pivotal role in inhibiting SC components from assembling into polycomplexes during these periods (Fig 10). As an E3 ubiquitin ligase, this role on SC formation is likely indirect. Sina likely targets for degradation a protein that promotes the assembly of SC components (both central region and LE proteins) into polycomplexes or inhibits normal assembly between the homologs. The localization of LE proteins to the *sina* polycomplexes could allow DNA association, including centromeres, with the polycomplexes. When both the LE protein C(2)M and Sina are absent, polycomplex formation is reduced, indicating that C(2)M further promotes polycomplex formation in *sina* mutants.

### What are the potential meiotic targets of Sina?

Since Sina proteins normally target other proteins for degradation, what is the likely target protein being degraded? One possibility is a protein that modifies the chromosome axis to prevent the loading and/or maintenance of SC components along the chromosomes. A second possibility is that Sina targets a protein that post-translationally modifies SC components to promote self-assembly into polycomplexes. In the premeiotic divisions of the 16-cell cyst, central region components are present and load to the centromeres but must be prevented from loading along the chromosome arms. Potentially, Sina could be degrading a meiotic regulator that helps to promote this pre-meiotic state of SC assembly. While overexpression of the ^*FLAG*^*sina*^*WT*^ construct rescued the meiotic phenotypes of *sina*^*A4*^*/sina*^*DF*^ females (S1 Fig), the resulting ^FLAG^Sina^WT^ protein recognized by an anti-FLAG antibody did not consistently localize to a cellular structure to suggest the potential target of Sina (S9A–S9C Fig). In addition, an anti-Sina antibody did not recognize any specific cellular structure in wild-type germaria (S9D Fig). This lack of localization of Sina to the SC supports the idea that Sina’s target is a protein that can modify SC assembly, rather than a SC component itself. Identifying this Sina target is an area of active research, as it will provide insight into determining what controls the decision of when and where to assemble full-length SC.

## Methods

### Stocks

The gene *seven in absentia* (*sina*, *CG9949*, FBgn0003410) is located on the *3*^*rd*^ chromosome. The *sina*^*A4*^ mutation is an A-to-V mutation at amino acid 270 that was isolated in an ethylmethanesulfonate-induced forward genetic screen. The mutagenized chromosome was recombined to pick up the visible markers *sr e ca* to remove linked mutations on *3R* resulting from the mutagenesis (see Flybase for descriptions). *sina*^*A4*^ refers to the genotype *sina*^*A4*^ *sr e ca*. The *sina*^*3*^ mutation is a deletion in the C-terminal half of the Sina protein that leads to an early stop at amino acid 212 and is presumed to be a null allele of *sina* [39]. The molecular lesion in the *sina*^*P21*^ allele has not been reported [43,44]. The deficiency *sinaSH* (named *sina*^*Df*^ here for simplicity) is a small deficiency removing parts of the *sina* and *sinaH* genes as described [68]. Wild type was *y w; spa*^*pol*^ except for experiments with genetic overexpression constructs and recombination. Overexpression constructs include *pUASp*: ^*3XHA*^*-c(2)M* (gift from Kim McKim) [9], *pUASP-Cona-venus* [7] and ^*FLAG*^*sina*^*WT*^ (described below). Overexpression constructs were driven in the germline by *Pnos-Gal4::VP16 [69]* located on the *X* chromosome.

### Immunohistochemistry

Imaging of early stages in whole-mount ovaries was carried out as described in [67] with two changes: female flies were yeasted and dissected 1–2 days post eclosion, and ovaries were dissected in PBS plus 0.1% TWEEN-20 (PBST).

For imaging of late-stage oocytes, ovaries were dissected in 1X Robb’s buffer (55 mM sodium acetate, 8 mM potassium acetate, 20 mM sucrose, 0.44 mM magnesium chloride, 0.01 mM calcium chloride, 20 mM HEPES) with 1% bovine serum albumin (BSA). Ovaries were fixed in 16% EM-grade formaldehyde (Ted Pella, Redding, CA) mixed 1:1 with a solution of 200 mM potassium cacodylate, 200 mM sucrose, 80 mM sodium acetate, and 20 mM EGTA for 5 min. Ovaries were washed 3 times in PBS plus 0.1% Triton X-100 (PBSTx) for at least 15 min each and then manually dechorionated between two frosted slides. Ovaries were blocked in PBSTx with 5% normal goat serum for at least 1 hr and then primary antibodies were left overnight in fresh blocking solution at 4°C. After 3 washes in PBSTx, secondary antibodies were applied in blocking solution for 4 hr with DAPI added during the last 15 min. After 3 washes in PBSTx, the samples were mounted in Prolog Gold (ThermoFisher Scientific).

Chromosome spreads were carried out as described in [48] with the few following minor changes. After later stages were removed in the hypo-buffer (30 mM Tris pH 8.0, 50 mM sucrose, 17 mM trisodium citrate dihydrate, 5 mM EDTA, 0.5 mM DTT, and 1X protease inhibitor cocktail (Sigma-Aldrich)), the ovary tips were transferred to 30 μl of 100 mM sucrose for fine mincing. The entire 30 μl of solution was spread along a single angled slide that had been dipped into fix (25 mL of a 1% paraformaldehyde solution with 350 μl Triton X-100) for 15 sec. For immunolocalization, the rehydrated slides were blocked in 5% goat serum, 2% BSA, 0.1% Triton X-100, 0.01% sodium azide in PBS under parafilm for 1 hr at room temperature in a humid chamber or overnight at 4°C. Primary antibodies were applied for 3 hr at room temperature or overnight at 4°C.

### Antibodies

Primary antibodies used for immunofluorescence include mouse anti-C(3)G 1A8-1G2, 5G4–1F1, and 1G5–2F7 (1:500 each) (1A8-1G2 was used for standard IF and immuno-EM, and a cocktail of all three C(3)G antibodies was used for STED) [12], affinity-purified rabbit anti-Corolla (animal 210) (1:2000) [8], guinea pig anti-Cona (1:500) [7], rabbit anti-Cid (1:2000) (gift of Dr. Gregory Rogers), rat anti-Cid (1:2000)[70], mouse anti-Orb antibodies 4H8 and 6H4 (1:20 each) (Developmental Studies Hybridoma Bank, Iowa), mouse anti-γH2AV (1:1000) [71], high-affinity rat anti-HA clone 3F10 (1:100) (Roche, Indianapolis, IN), rabbit anti-GFP (1:500) (AB6556, AbCam Inc.), mouse anti-lamin Dm0 ADL84.12 (1:100) (Developmental Studies Hybridoma Bank, Iowa), rat α-tubulin (1:250) (Serotec), mouse anti-FLAG (1:500) (F3165, Sigma-Aldrich), mouse anti-Siah2 (1:10) (24E6H3, Novus Biologicals), rat anti-Smc1 (1:250) (described below), and guinea pig anti-Smc3 (1:250) (described below).

Secondary goat anti-mouse, rabbit, guinea pig or rat Alexa-488, Alexa-555 and Alexa-647 IgG H&L chain conjugated antibodies (Molecular Probes,ThermoFisher Scientific) were used at 1:500 for early whole-mount samples and 1:400 for chromosome spreads and late stage-samples that used the DeltaVision and OMX microscopes. For STED, goat anti-mouse Abberior STAR 635 (Sigma Aldrich) and goat anti-rabbit Alexa-594 IgG H&L chain conjugated antibodies (Molecular Probes, ThermoFischer Scientific) were used at 1:500.

For the production of Smc1 and Smc3 antibodies, the following peptides were made by KLH using Biosynthesis: MTEEDDDVAQRVATAPVRKP-cys (Smc1) and CVTREEAKVFVEDDSTHA (Smc3). Peptides were then injected into a rat (Smc1) or guinea pig (Smc3) by Cocalico Biologicals.

### Microscopes and imaging conditions

A Deltavision Elite system from GE Healthcare equipped with an Olympus IX70 inverted microscope and a high-resolution CCD camera was used for most images. An Applied Precision OMX Blaze microscope (Issaquah, WA, USA) furnished with a PCO Edge sCMOS camera was used for standard super-resolution images. SoftWoRx v. 6.1 or 7.0.0 (Applied Precision/GE Healthcare) was used to deconvolve images (Deltavision and OMX) and reconstruct (OMX) images. SoftWoRx v. 6.1 or Imaris software 8.3.1 (Bitplane, Zurich, Switzerland) was used for image analysis. Brightness and contrast were adjusted minimally to visualize signals during figure preparation.

STED images were acquired with a Leica SP8 Gated STED microscope with a 100x, 1.4 N.A. objective. Abberior STAR 635 labelled probe was imaged with a pulsed white light (80 MHz) tuned to 635 nm; Alexa Fluor 594 labelled probe was imaged with the same white laser tuned at 594 nm. Both probes used a pulsed STED 775-nm laser as the depletion laser. All images were acquired in 2D mode with 80%–90% of maximum depletion laser power to maximize lateral resolution, and each image was averaged 8 times in line average mode. The emission photons were collected with an internal Leica HyD hybrid detector with a time gate between 1–6 ns. Raw STED images were deconvolved with the STED module in the Huygens professional deconvolution software (version 14.10; Scientific Volume Imaging). Deconvolution was processed with theoretical estimated point spread function and background from raw data, and the signal to noise was set in the range of 15–20 depending on the signal intensity. Other settings used were the system default.

### Immuno-EM

Whole-mount immuno-EM samples were prepared as described in [67]. Primary antibodies were mouse anti-C(3)G (1:500) and rabbit anti-Corolla (1:2000). Secondary antibodies used were anti-rabbit Alexa-488 and anti-mouse ultra-small gold (Electron Microscopy Sciences, Hatfield, PA). For on-section immunogold labeling, ultrathin sections of 50–70 nm in thickness were cut with a Diamond knife (DiATOME, PA), and mounted on Formvar-coated Nickle grids (EMS). The samples were incubated 1 hr at RT with the primary antibodies. After washing in PBS, samples were incubated for 1 hr with goat anti-mouse IgG conjugated to 6-nm gold particles and goat anti-rabbit IgG conjugated to 12-nm gold particles (Jackson ImmunoResearch Laboratories, PA). All sections were post-stained with uranyl acetate and lead citrate, observed, and imaged under a FEI transmission electron microscope.

### Quantification

For the quantification of polycomplexes in *sina*^*A4*^ homozygotes and *sina*^*A4*^*/sina*^*Df*^ ovaries images were scored that contained lamin antibody staining to demarcate the nuclei boundaries. Only nuclei with low optical distortion in z and minimal overlap with other SC positive nuclei were chosen for analysis. The number of polycomplexes (labeled with an antibody against Corolla) was scored by going through the entire nucleus defined by the lamin staining. Foci, puncta and track-like staining of central region components was excluded from the total polycomplex averages. For measurements of polycomplex size a subset of polycomplexes within the scored nuclei were analyzed that contained clearly defined ends for length measurements, showed low optical distortion, and were not too complex in shape (structures that differed in both length and width at multiple points were excluded for size measurements). For simple cylinder shapes the length and width were recorded. For cones, tapered ellipses, and other structures that changed in width, the width of the polycomplexes was taken at the widest point. For double mutants with the *c(2)M*^*EP2115*^ mutation nuclei were scored without the use of the lamin antibody. The decreased density of polycomplexes allowed for accurate scoring of polyocomplex number and size using only the C(3)G or Corolla antibodies and DAPI.

For analysis of γH2AV association, nuclei were examined by going through the z-stacks for each nuclei. A γH2AV focus was considered associated with a track of SC or a polycomplex if the γH2AV signal touched the Corolla signal or was within 0.2 of the nearest SC track or polycomplex.

### Hatch rate assays

Virgin females 1–3 days post eclosion of the indicated genotype were mated with wild type (*y w/y+ Y; spa*^*pol*^) males and allowed to acclimate in cages with grape plates supplemented with wet yeast paste for 1–2 days. Females were allowed to lay eggs on fresh grape plates with yeast paste for periods of 2–4 hr. The number of hatched and unhatched eggs was scored approximately 48 hr later. P-values were calculated using Fisher’s exact test in combination with a False Discovery Rate (FDR) correction for multiple testing.

### Meiotic nondisjunction and recombination assays

To measure the rate of both *X* and *4*^*th*^ chromosome nondisjunction, virgin females of the indicated genotype were mated to multiple *X^Y*, *In(1)EN*, *v f B; C(4)RM*, *ci ey*^*R*^ males. Assays of wild type (*y w; spa*^*pol*^) were done one female per vial, but due to the lower fertility of the *sina* mutants, two (*y; sina*^*A4*^*; spa*^*pol*^) or three (*y w/y; sina*^*A4*^*/sina*^*Df*^*; spa*^*pol*^) virgin females per vial were assayed to improve progeny viability. Calculations to determine the percentage of *X* and *4*^*th*^ chromosome nondisjunction were performed as previously described [72,73]. To calculate adjusted progeny in Table 1, the number of *X* chromosome exceptional progeny, as well as the number of *X+4*^*th*^ chromosome exceptional progeny, were doubled to account for inviable classes of progeny. This total was added to the total number of progeny with normal chromosome segregation and the number of *4*^*th*^ chromosome exceptional progeny to yield the final adjusted total of progeny.

To measure only *X* chromosome nondisjunction in S1B Fig and meiotic recombination along the *X* chromosome, virgin females (one virgin per vial for wild type, two for *sina*^*A4*^ and three for *sina*^*A4*^*/sina*^*Df*^ due to decreased fertility) were mated to *y sc cv v f/B*^*S*^*Y* males. For a description of the calculations of adjusted total progeny and *X* nondisjunction, see [66]. For recombination of the *X* chromosome in Table 5, genotypes were *y sc cv v f y+/y w* (wild type), *y sc cv v f y+/y; sina*^*A4*^, and *y sc cv v f y+/y w; sina*^*A4*^*/sina*^*Df*^. For recombination, only female progeny resulting from normal *X* chromosome segregation were scored for all markers.

### Construction of the FLAG-tagged wild-type *sina* rescue construct

The FLAG-tagged and *UASp*-driven wild-type *sina* rescue transgene was built by amplifying the *sina* coding region from cDNA using PCR while adding a 5’ *Not*I and a 3’*Xba*I restriction site. The fragment was subcloned into the *pUASp-attB-3XFLAG* vector [74] after digestion with *Not*I and *Xba*I. The construct was sequence verified and sent to Best Gene Inc. for injection using *ɸC31* site-specific integration into the *attP40* line.

### Protein alignment

For the alignment shown in Fig 1C the following sequences were obtained from UniProt: *D*. *melanogaster* Sina (P21461), *Homo Sapiens* Siah1 (Q8IUQ4), *Mus musculus* Siah1 (P61092), *Danio rerio* Siah1 (Q72VG6), *Xenopus laevis* Siah1 (Q6GQJ5) and *Hydra vulgaris* Siah1 (T2MA38). The protein domains are depicted as described in [16].

## Supporting information

S1 Fig Multiple phenotypes caused by the *sina*^*A4*^ mutation are rescued by overexpression of wild-type Sina protein. (A) Eggs from *sina*^*A4*^ and *sina*^*A4*^*/sina*^*Df*^ mothers hatch at a decreased rate, and the decreased hatch rate of *sina*^*A4*^*/sina*^*Df*^ mothers is rescued by overexpression of the ^*FLAG*^*sina*^*WT*^ construct driven by *Pnos-Gal4*::*VP16* in the germline. The percentage of hatched and unhatched eggs two days after deposition is shown. ** Denotes statistically significant from both wild-type and *nosGal4/+;* ^*FLAG*^*sina*^*WT*^*/+; sina*^*A4*^*/sina*^*Df*^ at P< 0.001. # Denotes statistical significance difference to *sina*^*A4*^ and *sina*^*A4*^*/sina*^*Df*^ at P< 0.001. While the ^*FLAG*^*sina*^*WT*^*/+; sina*^*A4*^*/sina*^*Df*^ line was statistically different than *sina*^*A4*^*/sina*^*Df*^ line, this difference is likely caused by genetic background differences. No other comparison was statistically significant. See Methods for statistical test. (B) Nondisjunction of the *X* chromosome in *sina*^*A4*^*/sina*^*Df*^ females was rescued by overexpression of ^*FLAG*^*sina*^*WT*^. **Statistically significant from wild type and *nosGal4/+;* ^*FLAG*^*sina*^*WT*^*/+; sina*^*A4*^*/sina*^*Df*^ at P< 0.001 based on the number of progeny scored. No other pair-wise comparison was statistically significant (P>0.05). Statistical test described in [40]. (C) The SC forms normally in a *sina* mutant germarium overexpressing ^*FLAG*^*sina*^*WT*^ (*nosGal4/+;* ^*FLAG*^*sina*^*WT*^*/+; sina*^*A4*^*/sina*^*Df*^) (bottom) compared to nuclei from a *sina* mutant germarium with the construct but without driver (^*FLAG*^*sina*^*WT*^*/+; sina*^*A4*^*/sina*^*Df*^) (top). C(3)G is labeled in yellow, DAPI in cyan. Scale bars, 1 μm. Images are projections of nuclei from larger *z*-stacks.
(TIF)

S2 Fig SC components form aberrant polycomplexes in *sina* mutants. (A) In wild-type germaria, track-like SC (Corolla in yellow) forms in multiple nuclei in early pachytene (region 2A, near top). As the cysts progress through the germarium, cells destined to be nurse cells disassemble their SC to leave a single pro-oocyte with SC at mid-pachytene (region 3) (yellow arrow). Orb (magenta) accumulates around the nucleus of the pro-oocyte by region 3 (magenta arrow). DAPI is in cyan. (B) In a *sina*^*A4*^ germarium, track-like SC can be observed in early pachytene (region 2A), but rod-like polycomplexes accumulate as the cysts progress through the germaria. While multiple nuclei have polycomplexes at mid-pachytene (region 3), Orb accumulates around a single nucleus (magenta/yellow arrows), demonstrating that persistence of polycomplexes in additional nuclei of region 3 (yellow arrowhead) is not due to an oocyte-specification problem. (C) In a *sina*^*A4*^*/sina*^*Df*^ germarium, aberrant SC polycomplexes of varying sizes can be observed even in early pachytene (region 2A). These aberrant SC structures persist not only in the oocyte nucleus designated with Orb (magenta/yellow arrows), but also in a nucleus destined to become a nurse cell in a cyst that is exiting region 3 (yellow arrowhead). Scale bar, 15 μm. (D) In wild-type nuclei in the premeiotic region 1 (top), Corolla (yellow) loads as foci before assembling along the chromosome arms in early pachytene (bottom of image). (E) In the premeiotic region 1 of the *sina*^*A4*^*/sina*^*Df*^ germarium, Corolla can be observed in small polycomplexes. DAPI is in cyan. Scale bar, 5 μm. Images are projections from *z*-stacks.
(TIF)

S3 Fig The length and width of polycomplexes in *sina* mutants by stage. The measurements of the length and width of polycomplexes from *sina*^*A4*^, *sina*^*A4*^*/sina*^*Df*^, *c(2)M*^*EP2115*^; *sina*^*A4*^ and *c(2)M*^*EP2115*^; *sina*^*A4*^*/sina*^*Df*^ females for the stages early pachytene (region 2A), early/mid-pachytene (region 2B), mid-pachytene (region 3), and mid-prophase (stages 2–9). See Fig 3 for graphs of combined data. Measurements are in microns.
(TIF)

S4 Fig Aberrant SC forms in other *sina* mutants. (A) An early/mid-pachytene nucleus from an ovary with a null allele of *sina* (*sina*^*3*^) in trans to the *sina*^*Df*^, with an approximately 5-μm polycomplex. (B-C) Nuclei from mothers that were *sina*^*3*^/*sina*^*A4*^ show aberrant SC during (B) early/mid-pachytene and (C) a stage 2 egg chamber with aberrant SC in both the oocyte nucleus (O) and three nurse cells (NC). (D) Polycomplexes form in *sina*^*P21*^/*sina*^*Df*^ germaria. C(3)G is in yellow and DAPI is in cyan. Scale bars, 1 μm (A, B, D) or 5 μm (C). Images are projections from larger *z*-stacks.
(TIF)

S5 Fig The central region component Cona localizes to *sina* polycomplexes. (A-C) Nuclei from whole-mount preparations of (A) wild type, (B) *sina*^*A4*^ and (C) *sina*^*A4*^*/sina*^*Df*^ labeled with C(3)G (yellow), Cona (magenta) and DAPI (cyan). Scale bars, 1 μm. (D) By SIM, Cona-Venus (GFP antibody in magenta) localizes between the two C-terminal tracks of C(3)G (green) in a *nosGAL4/+; cona-venus/+*; *cona*^*f04903*^/+ nucleus. (E) By SIM, the overexpressed Venus-tagged Cona construct alternates with the C-terminus of C(3)G in *sina*^*A4*^ polycomplexes (*nosGAL4/+; cona-venus/+; sina*^*A4*^). Scale bars, 1 μm. Images are partial projections from larger *z*-stacks.
(TIF)

S6 Fig Centromeres localize near the *sina* polycomplexes but centromere clustering is disrupted. (A–G) Nuclei labeled with DAPI (blue), Corolla (coral), Cid (yellow), and Lamin (cyan) to mark the nuclear envelope. (A) A wild-type nucleus with two centromere clusters associated with the SC. (B) Premeiotic nuclei (region 1) in wild type with Corolla localizing in foci associated with centromeres. (C-E) *sina*^*A4*^*/sina*^*Df*^ nuclei showing (C) centromere association with the small polycomplexes in region 1, (D) multiple centromeres associated with a large polycomplex, and (E) a single *z*-slice of a nucleus where centromere clustering could not be scored due to the centromere signal wrapping around a polycomplex rather than forming discrete foci. (F-G) *sina*^*A4*^ nuclei showing (F) wild-type-like centromere clustering in early pachytene (region 2A) when SC components form tracks and (G) small polycomplexes with multiple centromere clusters in mid-pachytene (region 3). Scale bars, 1 μm. Images are partial projections from larger *z*-stacks except where noted.
(TIF)

S7 Fig STED microscopy shows centromeres are associated with the *sina* polycomplexes. A side view of a polycomplex from a *sina*^*A4*^*/sina*^*Df*^ ovary showing centromeres (Cid, cyan) associated along the side of a large polycomplex (C(3)G, magenta). Scale bar, 1 μm. Image is a projection of a few *z*-slices.
(TIF)

S8 Fig Mutation of the LE protein *c(2)M* reduces polycomplex formation in *sina* mutants. (A–F) Germaria labeled with DAPI (cyan) and C(3)G (yellow), and oriented with the premeiotic region 1 at the bottom of each image and mid-pachytene (region 3) near the top. (A) wild-type germarium with tracks of SC. (B) *sina*^*A4*^ germarium with progressive formation of polycomplexes. (C) *sina*^*A4*^*/sina*^*Df*^ germarium displaying many polycomplexes. (D) *c(2)M*^*EP2115*^ germarium displaying only punctate SC. In *c(2)M*; *sina* double mutant nuclei (E–F), polycomplexes are still present but the number of polycomplexes within each nucleus is reduced compared to *sina* mutants alone. Scale bars, 15 μm. Images are projections of *z*-stacks.
(TIF)

S9 Fig Localization of the ^FLAG^sina^WT^ protein. While overexpression of the ^*FLAG*^*sina*^*WT*^ construct driven by *Pnos-Gal4*::*VP16* in the germline rescues *sina*^*A4*^*/sina*^*Df*^ phenotypes, the resulting ^FLAG^Sina^WT^ protein fails to show localization to specific cellular structures in germaria labeled with DAPI (cyan), Corolla (yellow) and FLAG (magenta). (A–B) Images from *nosGAL4/+;* ^*FLAG*^*sina*^*WT*^*/+; sina*^*A4*^*/sina*^*Df*^ germaria compared to (C) a germarium treated with FLAG antibody but lacking an expressed FLAG-tagged protein (*nosGAL4/+; sina*^*A4*^*/sina*^*Df*^). Left-hand panels show individual nuclei after deconvolution and projection of *z*-stacks while the right-hand images show a single *z*-slice from the raw images of whole germaria, demonstrating the lack of ^FLAG^Sina^WT^ localization is not due to a failure of expression. (D) Wild-type nuclei after deconvolution and projection of *z*-stacks.with a germarium before deconvolution showing the lack of localization of an antibody recognizing the N-terminus of Sina (magenta) with the central region component Corolla (yellow). DAPI is in cyan. Scale bars, 1 μm (nuclei), 15 μm (germaria).
(TIF)

S1 Table *X* and *4*^*th*^ chromosome nondisjunction frequency in *sina*^*A4*^*/sina*^*3*^ females. (DOCX)

## References

[1] Keeney S (2008) Spo11 and the Formation of DNA Double-Strand Breaks in Meiosis. Genome Dyn Stab 2: 81–123. 10.1007/7050_2007_026 21927624 PMC3172816

[2] Keeney S, Giroux CN, Kleckner N (1997) Meiosis-specific DNA double-strand breaks are catalyzed by Spo11, a member of a widely conserved protein family. Cell 88: 375–384. 9039264 10.1016/s0092-8674(00)81876-0

[3] Hughes SE, Miller DE, Miller AL, Hawley RS (2018) Female Meiosis: Synapsis, Recombination, and Segregation in Drosophila melanogaster. Genetics 208: 875–908. 10.1534/genetics.117.300081 29487146 PMC5844340

[4] McKim KS, Jang JK, Manheim EA (2002) Meiotic recombination and chromosome segregation in Drosophila females. Annu Rev Genet 36: 205–232. 10.1146/annurev.genet.36.041102.113929 12429692

[5] Gao J, Colaiacovo MP (2018) Zipping and Unzipping: Protein Modifications Regulating Synaptonemal Complex Dynamics. Trends Genet 34: 232–245. 10.1016/j.tig.2017.12.001 29290403 PMC5834363

[6] Page SL, Hawley RS (2001) *c(3)G* encodes a Drosophila synaptonemal complex protein. Genes Dev 15: 3130–3143. 10.1101/gad.935001 11731477 PMC312841

[7] Page SL, Khetani RS, Lake CM, Nielsen RJ, Jeffress JK, et al (2008) Corona is required for higher-order assembly of transverse filaments into full-length synaptonemal complex in Drosophila oocytes. PLoS Genet 4: e1000194 10.1371/journal.pgen.1000194 18802461 PMC2529403

[8] Collins KA, Unruh JR, Slaughter BD, Yu Z, Lake CM, et al (2014) Corolla is a novel protein that contributes to the architecture of the synaptonemal complex of Drosophila. Genetics 198: 219–228. 10.1534/genetics.114.165290 24913682 PMC4174934

[9] Manheim EA, McKim KS (2003) The synaptonemal complex component C(2)M regulates meiotic crossing over in Drosophila. Curr Biol 13: 276–285. 12593793 10.1016/s0960-9822(03)00050-2

[10] Takeo S, Lake CM, Morais-de-Sa E, Sunkel CE, Hawley RS (2011) Synaptonemal complex-dependent centromeric clustering and the initiation of synapsis in Drosophila oocytes. Curr Biol 21: 1845–1851. 10.1016/j.cub.2011.09.044 22036182

[11] Tanneti NS, Landy K, Joyce EF, McKim KS (2011) A pathway for synapsis initiation during zygotene in Drosophila oocytes. Curr Biol 21: 1852–1857. 10.1016/j.cub.2011.10.005 22036181 PMC12823172

[12] Anderson LK, Royer SM, Page SL, McKim KS, Lai A, et al (2005) Juxtaposition of C(2)M and the transverse filament protein C(3)G within the central region of Drosophila synaptonemal complex. Proc Natl Acad Sci U S A 102: 4482–4487. 10.1073/pnas.0500172102 15767569 PMC555515

[13] Moses MJ (1969) Structure and function of the synaptonemal complex. Genetics 61: Suppl:41–51. 5345399

[14] Carpenter AT (1975) Electron microscopy of meiosis in *Drosophila melanogaster* females. I. Structure, arrangement, and temporal change of the synaptonemal complex in wild-type. Chromosoma 51: 157–182. 806439 10.1007/BF00319833

[15] Grishaeva TM, Bogdanov YF (2014) Conservation and variability of synaptonemal complex proteins in phylogenesis of eukaryotes. Int J Evol Biol 2014: 856230 10.1155/2014/856230 25147749 PMC4132317

[16] Pepper IJ, Van Sciver RE, Tang AH (2017) Phylogenetic analysis of the SINA/SIAH ubiquitin E3 ligase family in Metazoa. BMC Evol Biol 17: 182 10.1186/s12862-017-1024-x 28784114 PMC5547486

[17] Cahoon CK, Yu Z, Wang Y, Guo F, Unruh JR, et al (2017) Superresolution expansion microscopy reveals the three-dimensional organization of the *Drosophila* synaptonemal complex Proc Natl Acad Sci U S A 114: E6857–E6866. 10.1073/pnas.1705623114 28760978 PMC5565445

[18] Gao J, Barroso C, Zhang P, Kim HM, Li S, et al (2016) N-terminal acetylation promotes synaptonemal complex assembly in C. elegans. Genes Dev 30: 2404–2416. 10.1101/gad.277350.116 27881602 PMC5131780

[19] Nadarajan S, Lambert TJ, Altendorfer E, Gao J, Blower MD, et al (2017) Polo-like kinase-dependent phosphorylation of the synaptonemal complex protein SYP-4 regulates double-strand break formation through a negative feedback loop. Elife 6. 10.7554/eLife.23437 PMC5423773 28346135

[20] Cheng CH, Lo YH, Liang SS, Ti SC, Lin FM, et al (2006) SUMO modifications control assembly of synaptonemal complex and polycomplex in meiosis of Saccharomyces cerevisiae. Genes Dev 20: 2067–2081. 10.1101/gad.1430406 16847351 PMC1536058

[21] Humphryes N, Leung WK, Argunhan B, Terentyev Y, Dvorackova M, et al (2013) The Ecm11-Gmc2 complex promotes synaptonemal complex formation through assembly of transverse filaments in budding yeast. PLoS Genet 9: e1003194 10.1371/journal.pgen.1003194 23326245 PMC3542071

[22] Rog O, Kohler S, Dernburg AF (2017) The synaptonemal complex has liquid crystalline properties and spatially regulates meiotic recombination factors. Elife 6. 10.7554/eLife.21455 PMC5268736 28045371

[23] Goldstein P (1987) Multiple synaptonemal complexes (polycomplexes): origin, structure and function. Cell Biol Int Rep 11: 759–796. 3319195 10.1016/0309-1651(87)90157-3

[24] Sym M, Roeder GS (1995) Zip1-induced changes in synaptonemal complex structure and polycomplex assembly. J Cell Biol 128: 455–466. 10.1083/jcb.128.4.455 7860625 PMC2199901

[25] Severson AF, Ling L, van Zuylen V, Meyer BJ (2009) The axial element protein HTP-3 promotes cohesin loading and meiotic axis assembly in C. elegans to implement the meiotic program of chromosome segregation. Genes Dev 23: 1763–1778. 10.1101/gad.1808809 19574299 PMC2720254

[26] Moses MJ (1968) Synaptinemal Complex. Annu Rev Genet 2: 363–412.

[27] Zickler D, Kleckner N (1999) Meiotic chromosomes: integrating structure and function. Annu Rev Genet 33: 603–754. 10.1146/annurev.genet.33.1.603 10690419

[28] Rasmussen SW (1975) Synaptonemal Polycomplexes in *Drosophila melanogaster*. Chromosoma 49 321–331.

[29] Severson AF, Meyer BJ (2014) Divergent kleisin subunits of cohesin specify mechanisms to tether and release meiotic chromosomes. Elife 3: e03467 10.7554/eLife.03467 25171895 PMC4174578

[30] Alleva B, Balukoff N, Peiper A, Smolikove S (2017) Regulating chromosomal movement by the cochaperone FKB-6 ensures timely pairing and synapsis. J Cell Biol 216: 393–408. 10.1083/jcb.201606126 28077446 PMC5294783

[31] Bowman R, Balukof N, Ford T, Smolikove S (2018) A Novel Role for alpha-importins and Akirin in Establishment of Meiotic Sister Chromatid Cohesion in Caenorhabditis elegans. Genetics. 10.1534/genetics.118.301458 PMC6366927 30563860

[32] Brockway H, Balukoff N, Dean M, Alleva B, Smolikove S (2014) The CSN/COP9 signalosome regulates synaptonemal complex assembly during meiotic prophase I of Caenorhabditis elegans. PLoS Genet 10: e1004757 10.1371/journal.pgen.1004757 25375142 PMC4222726

[33] Ollinger R, Alsheimer M, Benavente R (2005) Mammalian protein SCP1 forms synaptonemal complex-like structures in the absence of meiotic chromosomes. Mol Biol Cell 16: 212–217. 10.1091/mbc.E04-09-0771 15496453 PMC539165

[34] Hu, Zhang S, Vidal M, La Baer J, Xu T, et al (1997) Mammalian homologs of seven in absentia regulate DDC via the ubiquitin-proteasome pathway. Genes & Development 11: 2701–2714. 9334332 10.1101/gad.11.20.2701 PMC316613

[35] Zhang J, Guenther MG, Carthew RW, Lazar MA (1998) Proteasomal regulation of nuclear receptor corepressor-mediated repression. Genes Dev 12: 1775–1780. 10.1101/gad.12.12.1775 9637679 PMC316907

[36] Li S, Li Y, Carthew RW, Lai ZC (1997) Photoreceptor cell differentiation requires regulated proteolysis of the transcriptional repressor Tramtrack. Cell 90: 469–478. 9267027 10.1016/s0092-8674(00)80507-3

[37] Habelhah H, Frew IJ, Laine A, Janes PW, Relaix F, et al (2002) Stress-induced decrease in TRAF2 stability is mediated by Siah2. EMBO J 21: 5756–5765. 10.1093/emboj/cdf576 12411493 PMC131073

[38] Germani A, Bruzzoni-Giovanelli H, Fellous A, Gisselbrecht S, Varin-Blank N, et al (2000) SIAH-1 interacts with alpha-tubulin and degrades the kinesin Kid by the proteasome pathway during mitosis. Oncogene 19: 5997–6006. 10.1038/sj.onc.1204002 11146551

[39] Carthew RW, Rubin GM (1990) seven in absentia, a gene required for specification of R7 cell fate in the Drosophila eye. Cell 63: 561–577. 2146028 10.1016/0092-8674(90)90452-k

[40] Zeng Y, Li H, Schweppe NM, Hawley RS, Gilliland WD (2010) Statistical analysis of nondisjunction assays in Drosophila. Genetics 186: 505–513. 10.1534/genetics.110.118778 20660647 PMC2954469

[41] Christophorou N, Rubin T, Huynh JR (2013) Synaptonemal complex components promote centromere pairing in pre-meiotic germ cells. PLoS Genet 9: e1004012 10.1371/journal.pgen.1004012 24367278 PMC3868581

[42] Carthew RW, Neufeld TP, Rubin GM (1994) Identification of genes that interact with the sina gene in Drosophila eye development. Proc Natl Acad Sci U S A 91: 11689–11693. 10.1073/pnas.91.24.11689 7972125 PMC45297

[43] Begemann G, Michon AM, vd Voorn L, Wepf R, Mlodzik M (1995) The Drosophila orphan nuclear receptor seven-up requires the Ras pathway for its function in photoreceptor determination. Development 121: 225–235. 7867504 10.1242/dev.121.1.225

[44] Mlodzik M, Hiromi Y, Goodman CS, Rubin GM (1992) The presumptive R7 cell of the developing Drosophila eye receives positional information independent of sevenless, boss and sina. Mech Dev 37: 37–42. 1606018 10.1016/0925-4773(92)90013-a

[45] Jeffress JK, Page SL, Royer SK, Belden ED, Blumenstiel JP, et al (2007) The formation of the central element of the synaptonemal complex may occur by multiple mechanisms: the roles of the N- and C-terminal domains of the Drosophila C(3)G protein in mediating synapsis and recombination. Genetics 177: 2445–2456. 10.1534/genetics.107.078717 17947423 PMC2219479

[46] Afshar K, Barton NR, Hawley RS, Goldstein LS (1995) DNA binding and meiotic chromosomal localization of the *Drosophila* nod kinesin-like protein. Cell 81: 129–138. 7720068 10.1016/0092-8674(95)90377-1

[47] Dong H, Roeder GS (2000) Organization of the yeast Zip1 protein within the central region of the synaptonemal complex. J Cell Biol 148: 417–426. 10.1083/jcb.148.3.417 10662769 PMC2174805

[48] Khetani RS, Bickel SE (2007) Regulation of meiotic cohesion and chromosome core morphogenesis during pachytene in Drosophila oocytes. J Cell Sci 120: 3123–3137. 10.1242/jcs.009977 17698920

[49] Lantz V, Ambrosio L, Schedl P (1992) The Drosophila orb gene is predicted to encode sex-specific germline RNA-binding proteins and has localized transcripts in ovaries and early embryos. Development 115: 75–88. 1638994 10.1242/dev.115.1.75

[50] Lantz V, Chang JS, Horabin JI, Bopp D, Schedl P (1994) The Drosophila orb RNA-binding protein is required for the formation of the egg chamber and establishment of polarity. Genes Dev 8: 598–613. 10.1101/gad.8.5.598 7523244

[51] Blower MD, Karpen GH (2001) The role of Drosophila CID in kinetochore formation, cell-cycle progression and heterochromatin interactions. Nat Cell Biol 3: 730–739. 10.1038/35087045 11483958 PMC3229202

[52] Mehrotra S, McKim KS (2006) Temporal analysis of meiotic DNA double-strand break formation and repair in Drosophila females. PLoS Genet 2: e200 10.1371/journal.pgen.0020200 17166055 PMC1657055

[53] Jang JK, Messina L, Erdman MB, Arbel T, Hawley RS (1995) Induction of metaphase arrest in Drosophila oocytes by chiasma-based kinetochore tension. Science 268: 1917–1919. 7604267 10.1126/science.7604267

[54] Lake CM, Nielsen RJ, Hawley RS (2011) The Drosophila zinc finger protein *trade embargo* is required for double strand break formation in meiosis. PLoS Genet 7: e1002005 10.1371/journal.pgen.1002005 21383963 PMC3044681

[55] Gyuricza MR, Manheimer KB, Apte V, Krishnan B, Joyce EF, et al (2016) Dynamic and stable cohesins regulate synaptonemal complex assembly and chromosome segregation. Curr Biol 26: 1688–1698. 10.1016/j.cub.2016.05.006 27291057 PMC4942336

[56] Ahuja JS, Sandhu R, Mainpal R, Lawson C, Henley H, et al (2017) Control of meiotic pairing and recombination by chromosomally tethered 26S proteasome. Science 355: 408–411. 10.1126/science.aaf4778 28059715 PMC6054871

[57] Rao HB, Qiao H, Bhatt SK, Bailey LR, Tran HD, et al (2017) A SUMO-ubiquitin relay recruits proteasomes to chromosome axes to regulate meiotic recombination. Science 355: 403–407. 10.1126/science.aaf6407 28059716 PMC5569317

[58] Dickins RA, Frew IJ, House CM, O'Bryan MK, Holloway AJ, et al (2002) The ubiquitin ligase component Siah1a is required for completion of meiosis I in male mice. Mol Cell Biol 22: 2294–2303. 10.1128/MCB.22.7.2294-2303.2002 11884614 PMC133675

[59] Polekhina G, House CM, Traficante N, Mackay JP, Relaix F, et al (2002) Siah ubiquitin ligase is structurally related to TRAF and modulates TNF-alpha signaling. Nat Struct Biol 9: 68–75. 10.1038/nsb743 11742346

[60] Rimsa V, Eadsforth TC, Hunter WN (2013) Two high-resolution structures of the human E3 ubiquitin ligase Siah1. Acta Crystallogr Sect F Struct Biol Cryst Commun 69: 1339–1343. 10.1107/S1744309113031448 24316825 PMC3855715

[61] Tang AH, Neufeld TP, Kwan E, Rubin GM (1997) PHYL acts to down-regulate TTK88, a transcriptional repressor of neuronal cell fates, by a SINA-dependent mechanism. Cell 90: 459–467. 9267026 10.1016/s0092-8674(00)80506-1

[62] Nagano Y, Fukushima T, Okemoto K, Tanaka K, Bowtell DD, et al (2011) Siah1/SIP regulates p27(kip1) stability and cell migration under metabolic stress. Cell Cycle 10: 2592–2602. 10.4161/cc.10.15.16912 21734459 PMC3180198

[63] Pi H, Wu HJ, Chien CT (2001) A dual function of phyllopod in Drosophila external sensory organ development: cell fate specification of sensory organ precursor and its progeny. Development 128: 2699–2710. 11526076 10.1242/dev.128.14.2699

[64] Bhuiyan H, Dahlfors G, Schmekel K (2003) Lateral elements inside synaptonemal complex-like polycomplexes in ndt80 mutants of yeast bind DNA. Genetics 163: 539–544. 12618393 10.1093/genetics/163.2.539 PMC1462438

[65] Tsubouchi T, Macqueen AJ, Roeder GS (2008) Initiation of meiotic chromosome synapsis at centromeres in budding yeast. Genes Dev 22: 3217–3226. 10.1101/gad.1709408 19056898 PMC2593611

[66] Lake CM, Nielsen RJ, Bonner AM, Eche S, White-Brown S, et al (2019) Narya, a RING finger domain-containing protein, is required for meiotic DNA double-strand break formation and crossover maturation in Drosophila melanogaster. PLoS Genet 15: e1007886 10.1371/journal.pgen.1007886 30615609 PMC6336347

[67] Lake CM, Nielsen RJ, Guo F, Unruh JR, Slaughter BD, et al (2015) Vilya, a component of the recombination nodule, is required for meiotic double-strand break formation in Drosophila. Elife 4: e08287 10.7554/eLife.08287 26452093 PMC4703084

[68] Cooper SE (2007) In vivo function of a novel Siah protein in Drosophila. Mech Dev 124: 584–591. 10.1016/j.mod.2007.04.007 17561381

[69] Van Doren M, Williamson AL, Lehmann R (1998) Regulation of zygotic gene expression in Drosophila primordial germ cells. Curr Biol 8: 243–246. 9501989 10.1016/s0960-9822(98)70091-0

[70] Hanlon SL, Miller DE, Eche S, Hawley RS (2018) Origin, Composition, and Structure of the Supernumerary B Chromosome of Drosophila melanogaster. Genetics 210: 1197–1212. 10.1534/genetics.118.301478 30249684 PMC6283169

[71] Lake CM, Holsclaw JK, Bellendir SP, Sekelsky J, Hawley RS (2013) The development of a monoclonal antibody recognizing the *Drosophila melanogaster* phosphorylated histone H2A variant (gamma-H2AV). G3 (Bethesda) 3: 1539–1543. 23833215 10.1534/g3.113.006833 PMC3755914

[72] Zitron AE, Hawley RS (1989) The genetic analysis of distributive segregation in *Drosophila melanogaster*. I. Isolation and characterization of *Aberrant X segregation* (*Axs*), a mutation defective in chromosome partner choice. Genetics 122: 801–821. 2503421 10.1093/genetics/122.4.801 PMC1203756

[73] Hawley RS, Irick H, Zitron AE, Haddox DA, Lohe A, et al (1992) There are two mechanisms of achiasmate segregation in Drosophila females, one of which requires heterochromatic homology. Dev Genet 13: 440–467. 10.1002/dvg.1020130608 1304424

[74] Bonner AM, Hughes SE, Chisholm JA, Smith SK, Slaughter BD, et al (2013) Binding of *Drosophila* Polo kinase to its regulator Matrimony is noncanonical and involves two separate functional domains. Proc Natl Acad Sci U S A 110: E1222–1231. 10.1073/pnas.1301690110 23479640 PMC3612667

