# Supplementary figures and images for: The E3 ubiquitin ligase Sina regulates the assembly and disassembly of the synaptonemal complex in Drosophila females
Source: PLoS Genet. 2019 May 20;15(5):e1008161. doi: 10.1371/journal.pgen.1008161 (PMC6544331; doi:10.1371/journal.pgen.1008161)

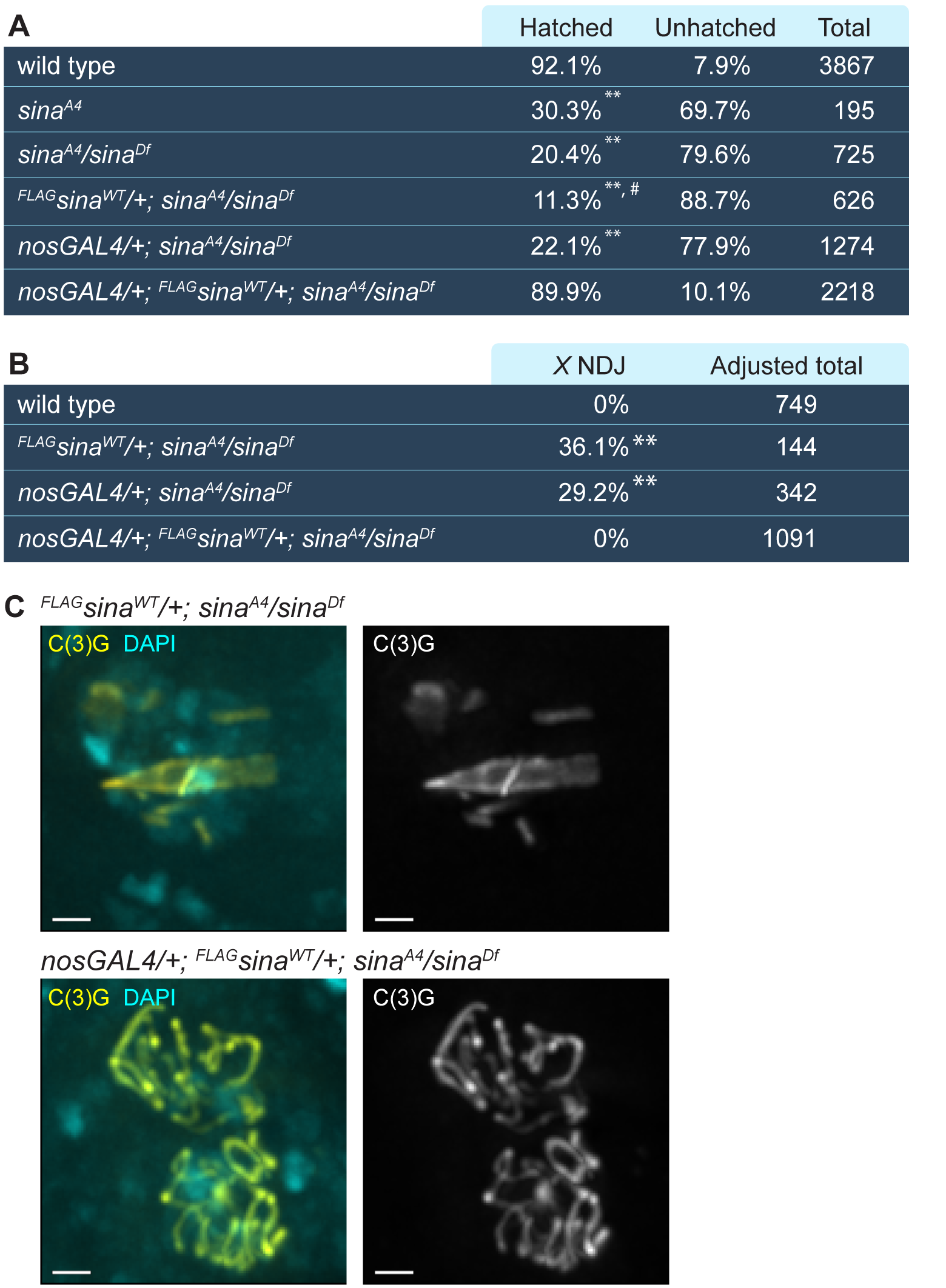

Supplement: S1 Fig — (A) Eggs from sinaA4 and sinaA4/sinaDf mothers hatch at a decreased rate, and the decreased hatch rate of sinaA4/sinaDf mothers is rescued by overexpression of the FLAGsinaWT construct driven by Pnos-Gal4::VP16 in the germline. The percentage of hatched and unhatched eggs two days after deposition is shown. ** Denotes statistically significant from both wild-type and nosGal4/+; FLAGsinaWT/+; sinaA4/sinaDf at P< 0.001. # Denotes statistical significance difference to sinaA4 and sinaA4/sinaDf at P< 0.001. While the FLAGsinaWT/+; sinaA4/sinaDf line was statistically different than sinaA4/sinaDf line, this difference is likely caused by genetic background differences. No other comparison was statistically significant. See Methods for statistical test. (B) Nondisjunction of the X chromosome in sinaA4/sinaDf females was rescued by overexpression of FLAGsinaWT. **Statistically significant from wild type and nosGal4/+; FLAGsinaWT/+; sinaA4/sinaDf at P< 0.001 based on the number of progeny scored. No other pair-wise comparison was statistically significant (P>0.05). Statistical test described in [40]. (C) The SC forms normally in a sina mutant germarium overexpressing FLAGsinaWT (nosGal4/+; FLAGsinaWT/+; sinaA4/sinaDf) (bottom) compared to nuclei from a sina mutant germarium with the construct but without driver (FLAGsinaWT/+; sinaA4/sinaDf) (top). C(3)G is labeled in yellow, DAPI in cyan. Scale bars, 1 μm. Images are projections of nuclei from larger z-stacks. (TIF) [file pgen.1008161.s001.tif]

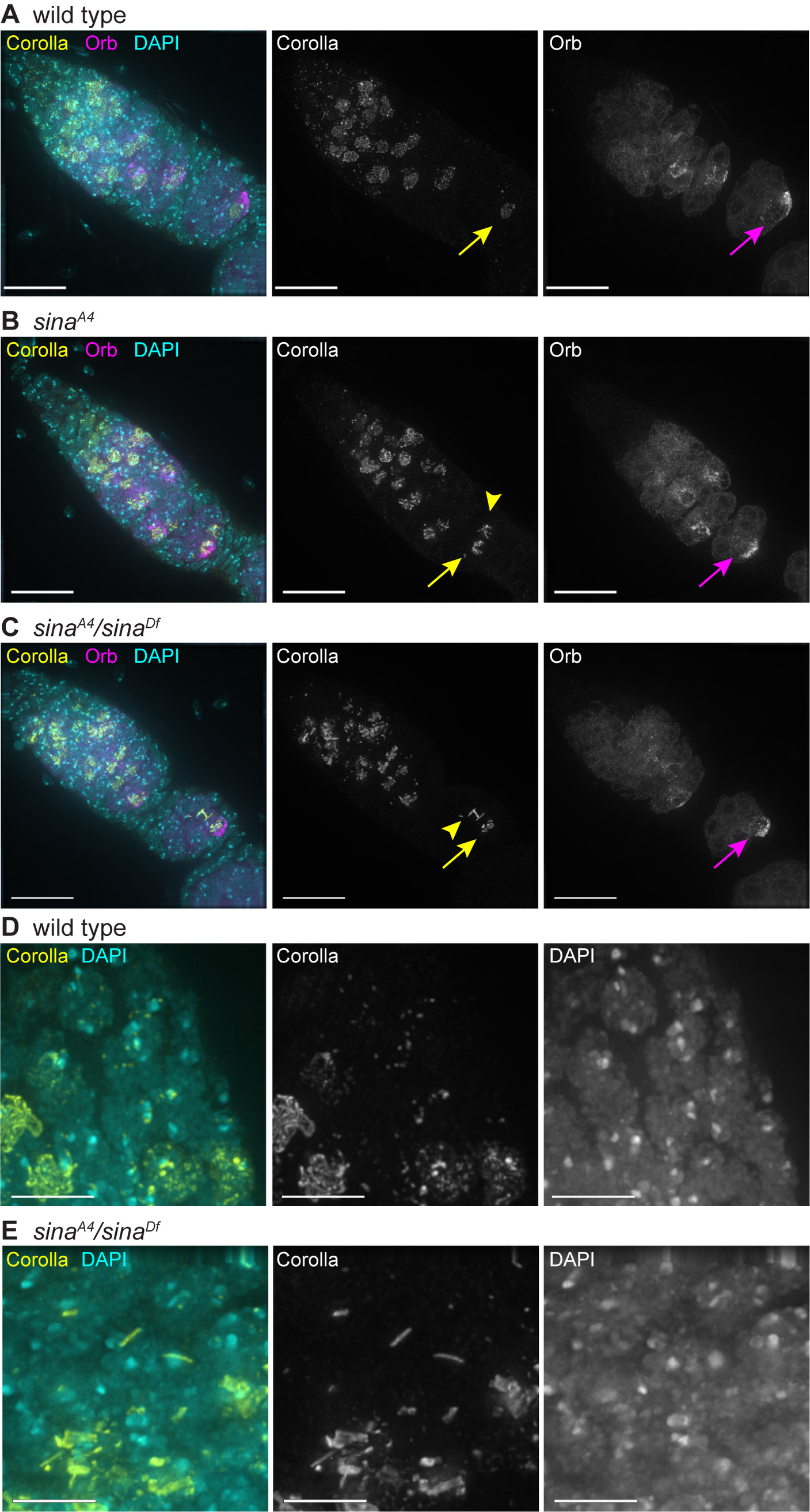

Supplement: S2 Fig — (A) In wild-type germaria, track-like SC (Corolla in yellow) forms in multiple nuclei in early pachytene (region 2A, near top). As the cysts progress through the germarium, cells destined to be nurse cells disassemble their SC to leave a single pro-oocyte with SC at mid-pachytene (region 3) (yellow arrow). Orb (magenta) accumulates around the nucleus of the pro-oocyte by region 3 (magenta arrow). DAPI is in cyan. (B) In a sinaA4 germarium, track-like SC can be observed in early pachytene (region 2A), but rod-like polycomplexes accumulate as the cysts progress through the germaria. While multiple nuclei have polycomplexes at mid-pachytene (region 3), Orb accumulates around a single nucleus (magenta/yellow arrows), demonstrating that persistence of polycomplexes in additional nuclei of region 3 (yellow arrowhead) is not due to an oocyte-specification problem. (C) In a sinaA4/sinaDf germarium, aberrant SC polycomplexes of varying sizes can be observed even in early pachytene (region 2A). These aberrant SC structures persist not only in the oocyte nucleus designated with Orb (magenta/yellow arrows), but also in a nucleus destined to become a nurse cell in a cyst that is exiting region 3 (yellow arrowhead). Scale bar, 15 μm. (D) In wild-type nuclei in the premeiotic region 1 (top), Corolla (yellow) loads as foci before assembling along the chromosome arms in early pachytene (bottom of image). (E) In the premeiotic region 1 of the sinaA4/sinaDf germarium, Corolla can be observed in small polycomplexes. DAPI is in cyan. Scale bar, 5 μm. Images are projections from z-stacks. (TIF) [file pgen.1008161.s002.tif]

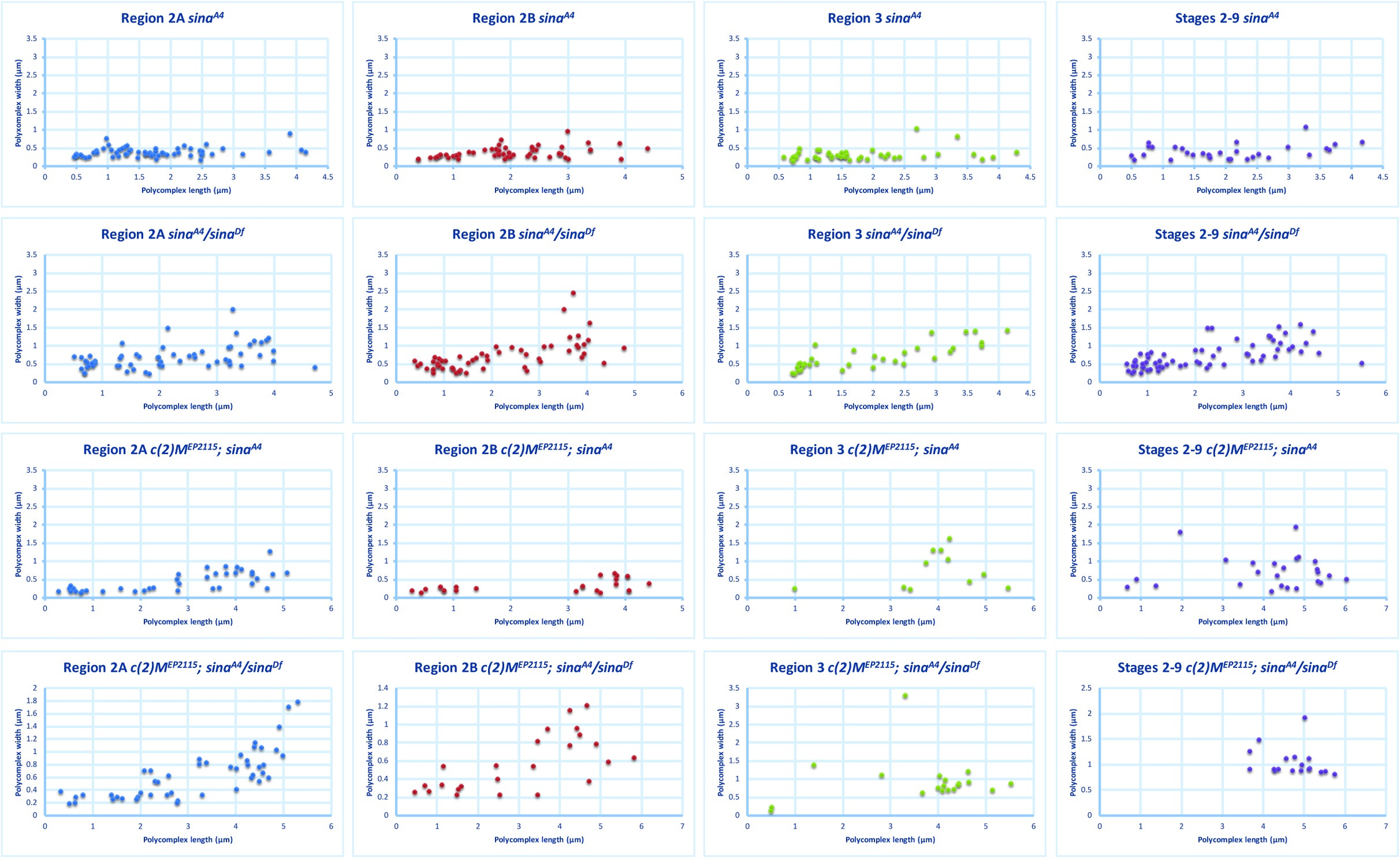

Supplement: S3 Fig — The measurements of the length and width of polycomplexes from sinaA4, sinaA4/sinaDf, c(2)MEP2115; sinaA4 and c(2)MEP2115; sinaA4/sinaDf females for the stages early pachytene (region 2A), early/mid-pachytene (region 2B), mid-pachytene (region 3), and mid-prophase (stages 2–9). See Fig 3 for graphs of combined data. Measurements are in microns. (TIF) [file pgen.1008161.s003.tif]

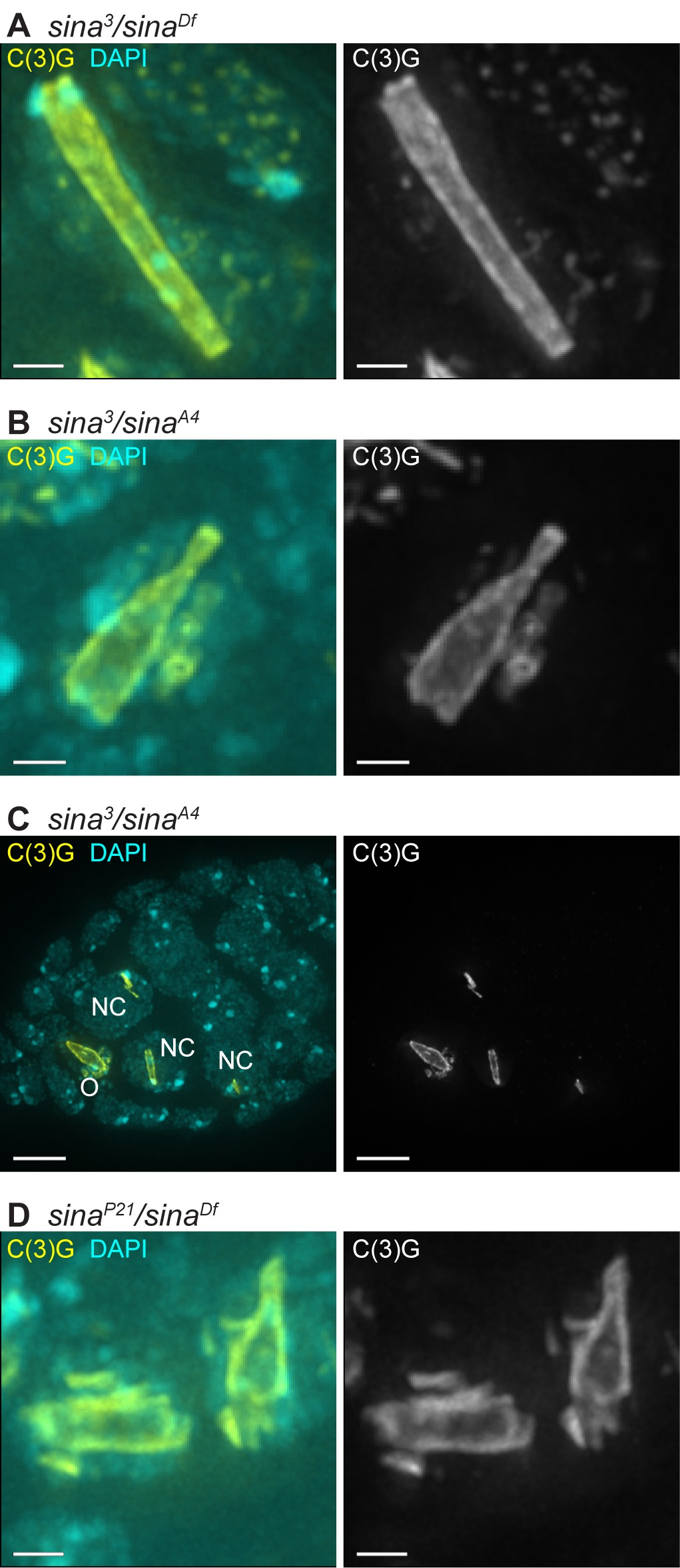

Supplement: S4 Fig — (A) An early/mid-pachytene nucleus from an ovary with a null allele of sina (sina3) in trans to the sinaDf, with an approximately 5-μm polycomplex. (B-C) Nuclei from mothers that were sina3/sinaA4 show aberrant SC during (B) early/mid-pachytene and (C) a stage 2 egg chamber with aberrant SC in both the oocyte nucleus (O) and three nurse cells (NC). (D) Polycomplexes form in sinaP21/sinaDf germaria. C(3)G is in yellow and DAPI is in cyan. Scale bars, 1 μm (A, B, D) or 5 μm (C). Images are projections from larger z-stacks. (TIF) [file pgen.1008161.s004.tif]

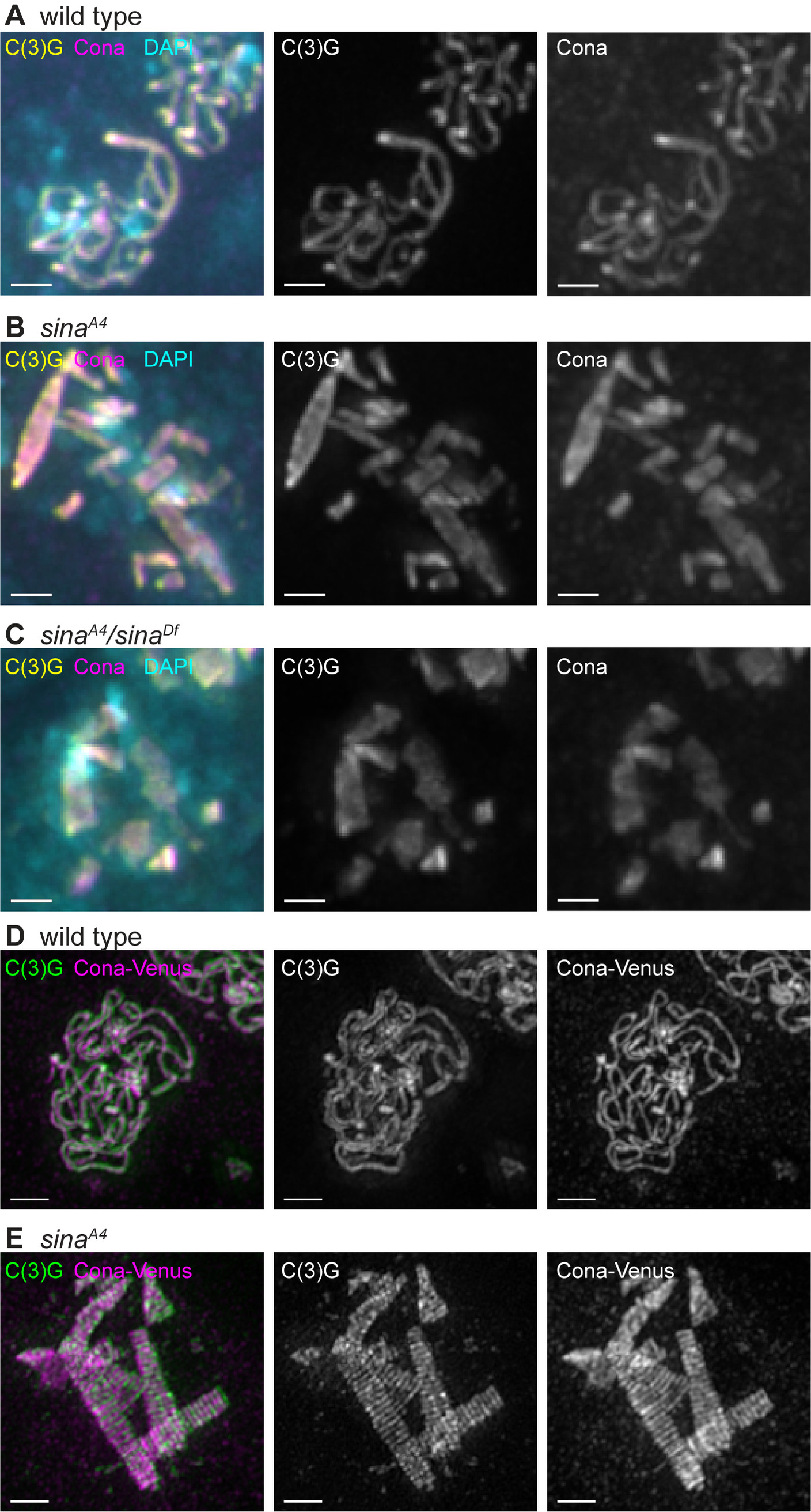

Supplement: S5 Fig — (A-C) Nuclei from whole-mount preparations of (A) wild type, (B) sinaA4 and (C) sinaA4/sinaDf labeled with C(3)G (yellow), Cona (magenta) and DAPI (cyan). Scale bars, 1 μm. (D) By SIM, Cona-Venus (GFP antibody in magenta) localizes between the two C-terminal tracks of C(3)G (green) in a nosGAL4/+; cona-venus/+; conaf04903/+ nucleus. (E) By SIM, the overexpressed Venus-tagged Cona construct alternates with the C-terminus of C(3)G in sinaA4 polycomplexes (nosGAL4/+; cona-venus/+; sinaA4). Scale bars, 1 μm. Images are partial projections from larger z-stacks. (TIF) [file pgen.1008161.s005.tif]

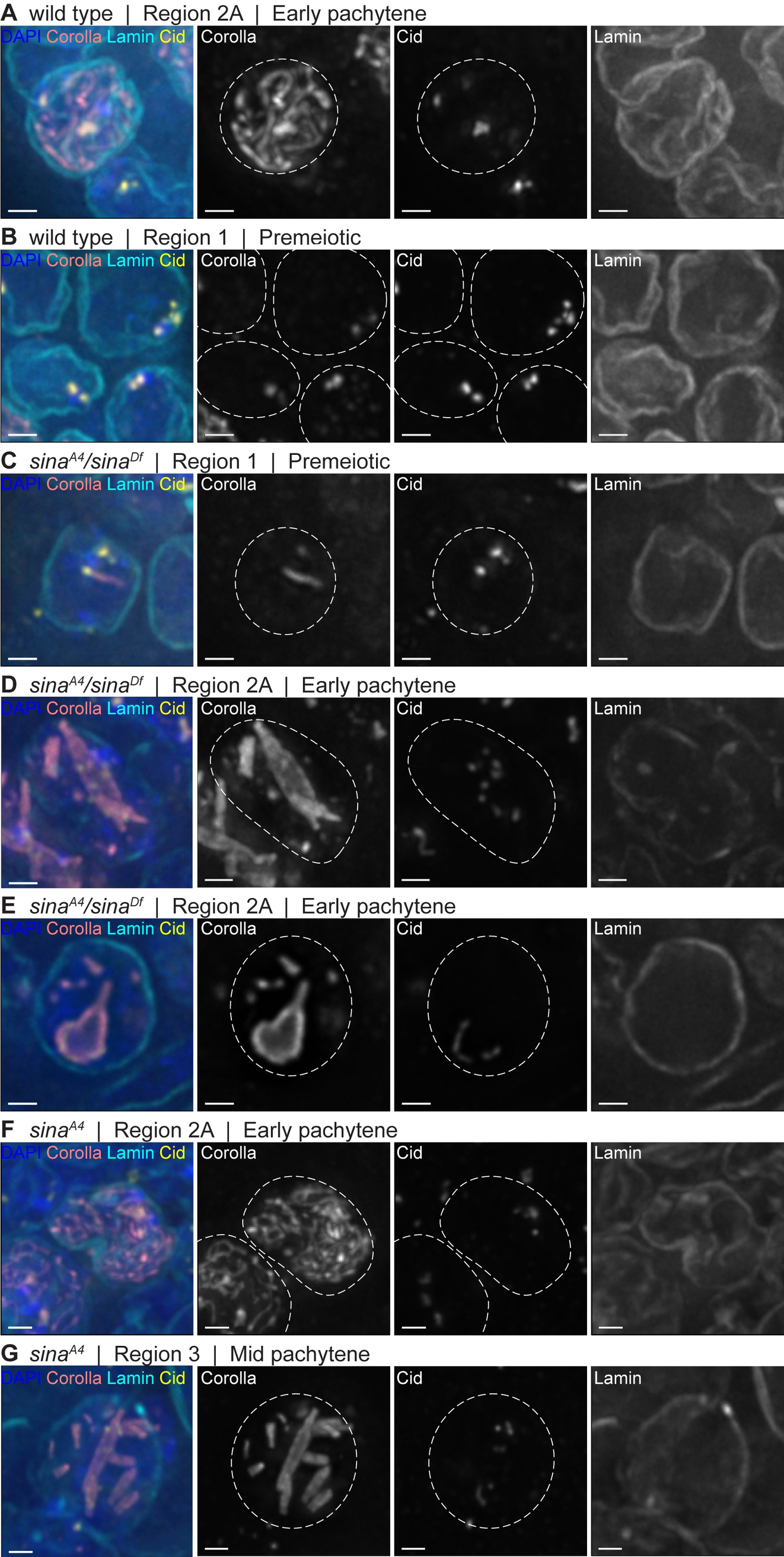

Supplement: S6 Fig — (A–G) Nuclei labeled with DAPI (blue), Corolla (coral), Cid (yellow), and Lamin (cyan) to mark the nuclear envelope. (A) A wild-type nucleus with two centromere clusters associated with the SC. (B) Premeiotic nuclei (region 1) in wild type with Corolla localizing in foci associated with centromeres. (C-E) sinaA4/sinaDf nuclei showing (C) centromere association with the small polycomplexes in region 1, (D) multiple centromeres associated with a large polycomplex, and (E) a single z-slice of a nucleus where centromere clustering could not be scored due to the centromere signal wrapping around a polycomplex rather than forming discrete foci. (F-G) sinaA4 nuclei showing (F) wild-type-like centromere clustering in early pachytene (region 2A) when SC components form tracks and (G) small polycomplexes with multiple centromere clusters in mid-pachytene (region 3). Scale bars, 1 μm. Images are partial projections from larger z-stacks except where noted. (TIF) [file pgen.1008161.s006.tif]

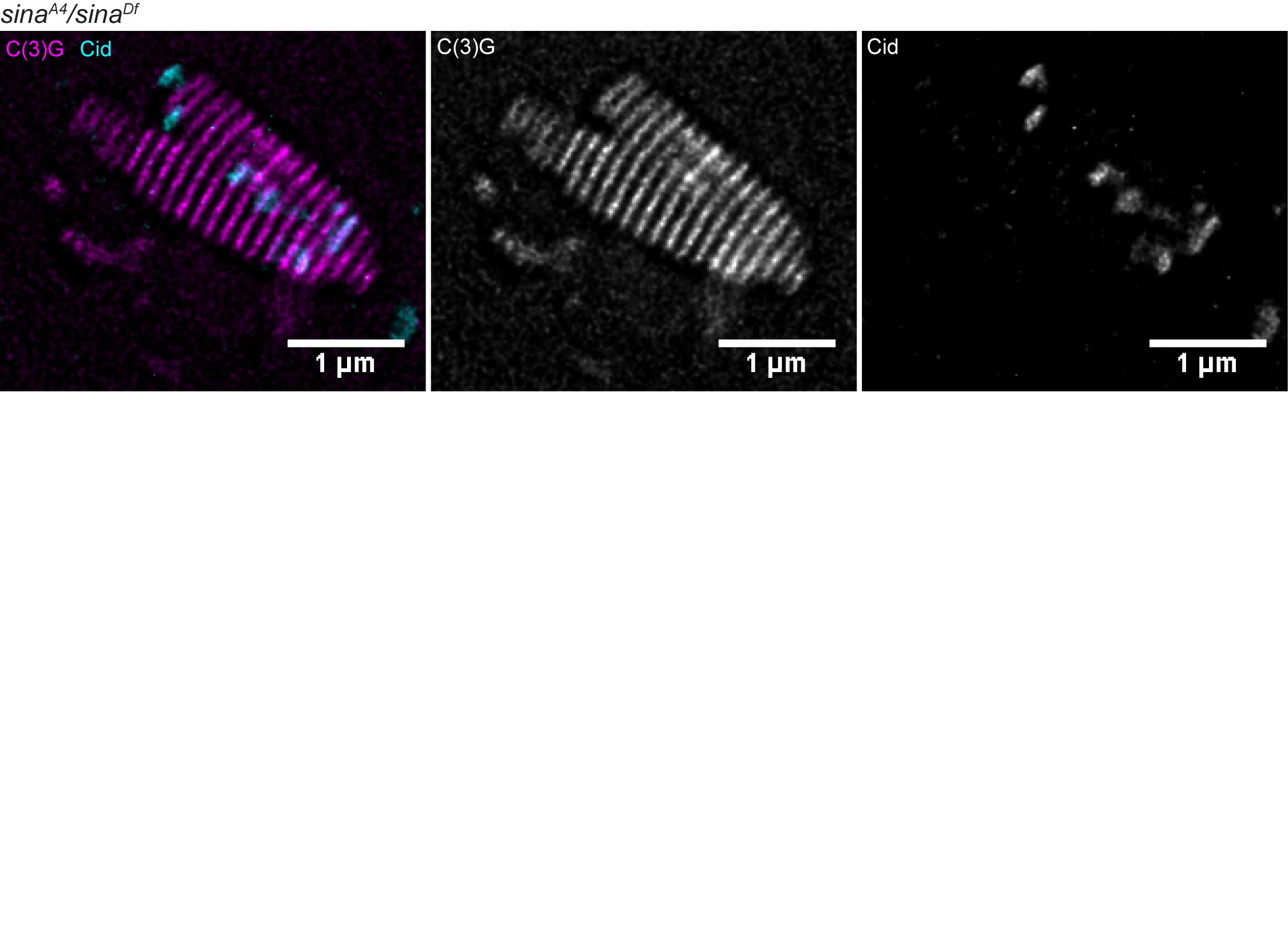

Supplement: S7 Fig — A side view of a polycomplex from a sinaA4/sinaDf ovary showing centromeres (Cid, cyan) associated along the side of a large polycomplex (C(3)G, magenta). Scale bar, 1 μm. Image is a projection of a few z-slices. (TIF) [file pgen.1008161.s007.tif]

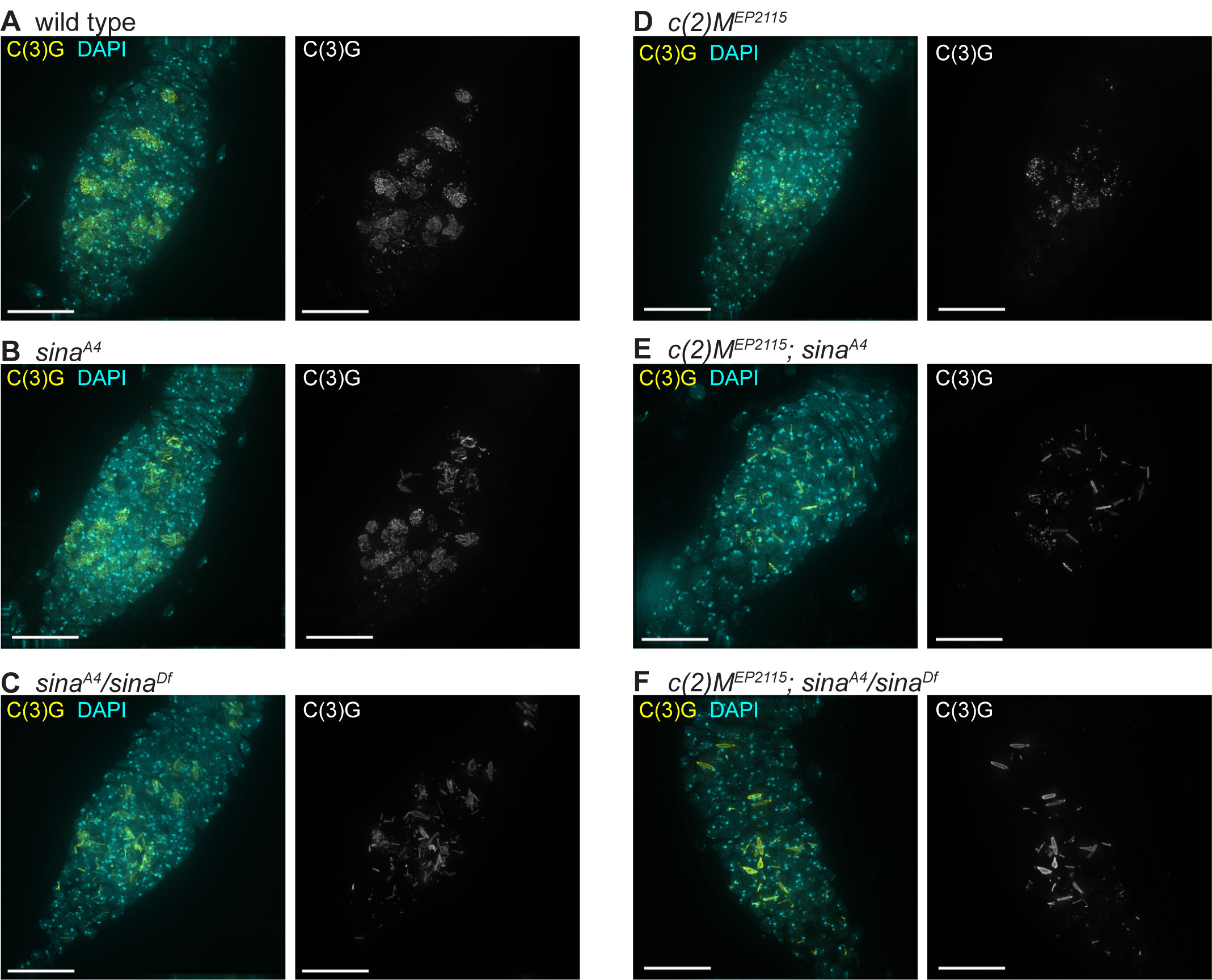

Supplement: S8 Fig — (A–F) Germaria labeled with DAPI (cyan) and C(3)G (yellow), and oriented with the premeiotic region 1 at the bottom of each image and mid-pachytene (region 3) near the top. (A) wild-type germarium with tracks of SC. (B) sinaA4 germarium with progressive formation of polycomplexes. (C) sinaA4/sinaDf germarium displaying many polycomplexes. (D) c(2)MEP2115 germarium displaying only punctate SC. In c(2)M; sina double mutant nuclei (E–F), polycomplexes are still present but the number of polycomplexes within each nucleus is reduced compared to sina mutants alone. Scale bars, 15 μm. Images are projections of z-stacks. (TIF) [file pgen.1008161.s008.tif]

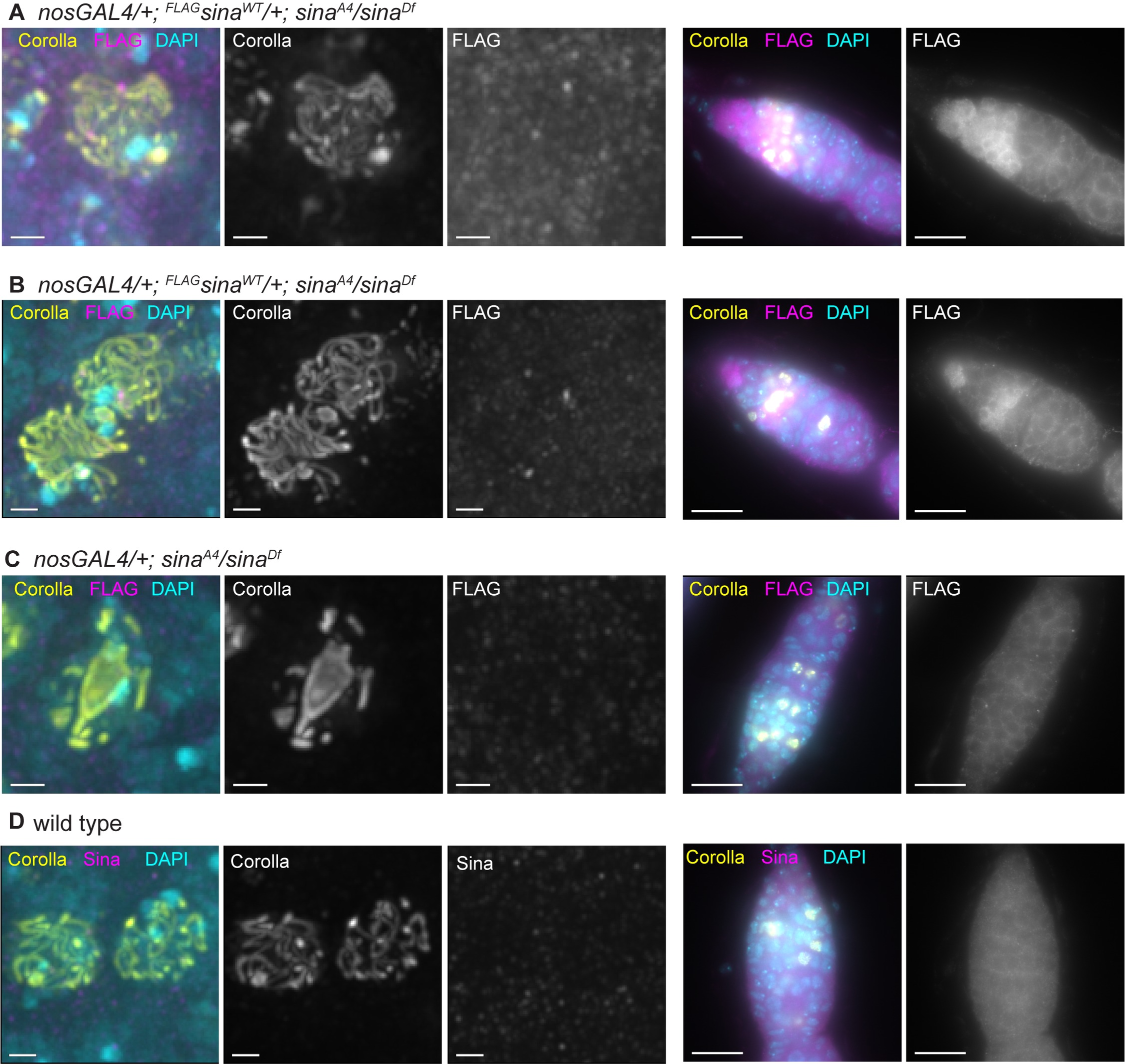

Supplement: S9 Fig — While overexpression of the FLAGsinaWT construct driven by Pnos-Gal4::VP16 in the germline rescues sinaA4/sinaDf phenotypes, the resulting FLAGSinaWT protein fails to show localization to specific cellular structures in germaria labeled with DAPI (cyan), Corolla (yellow) and FLAG (magenta). (A–B) Images from nosGAL4/+; FLAGsinaWT/+; sinaA4/sinaDf germaria compared to (C) a germarium treated with FLAG antibody but lacking an expressed FLAG-tagged protein (nosGAL4/+; sinaA4/sinaDf). Left-hand panels show individual nuclei after deconvolution and projection of z-stacks while the right-hand images show a single z-slice from the raw images of whole germaria, demonstrating the lack of FLAGSinaWT localization is not due to a failure of expression. (D) Wild-type nuclei after deconvolution and projection of z-stacks.with a germarium before deconvolution showing the lack of localization of an antibody recognizing the N-terminus of Sina (magenta) with the central region component Corolla (yellow). DAPI is in cyan. Scale bars, 1 μm (nuclei), 15 μm (germaria). (TIF) [file pgen.1008161.s009.tif]
