# Supplementary material for: The E3 ubiquitin ligase Sina regulates the assembly and disassembly of the synaptonemal complex in Drosophila females
Source: PLoS Genet. 2019 May 20;15(5):e1008161. doi: 10.1371/journal.pgen.1008161 (PMC6544331; doi:10.1371/journal.pgen.1008161)
Supplement: S1 Table — (DOCX) [file pgen.1008161.s010.docx]

**S1 Table.** *X* and *4^th^* chromosome nondisjunction frequency in *sina^A4^/sina^3^* females

| Gamete Type | |  |  |
| --- | --- | --- | --- |
| Maternal | Paternal | Wild type^a^ | *sina^A4/^sina^3^* |
| *X;4* | *XY; 44* | 317 | 62 |
| *X;4* | *0; 44* | 398 | 71 |
| *X* NDJ^b^ |  |  |  |
| *0; 4* | *XY; 44* | 2 | 21 |
| *XX; 4* | *0; 44* | 0 | 36 |
| *4* NDJ |  |  |  |
| *X; 0* | *XY; 44* | 0 | 19 |
| X; 0 | *0; 44* | 0 | 12 |
| *X; 44* | *XY; 0* | 0 | 8 |
| *X; 44* | *0; 0* | 0 | 8 |
| *X; 4* NDJ |  |  |  |
| *0; 0* | *XY; 44* | 0 | 24 |
| *XX; 44* | *0; 0* | 0 | 14 |
| *0; 44* | *XY; 0* | 0 | 2 |
| *XX; 0* | *0; 44* | 0 | 2 |
| Total Progeny |  | 717 | 279 |
| Adjusted Progeny^c^ |  | 719 | 378 |
| % X NDJ |  | 0.6 | 52.4** |
| % 4 NDJ |  | 0.0 | 34.7** |
| % nullo-*X* |  | 0.6 | 24.9 |
| % diplo-*X* |  | 0.0 | 27.5 |
| % nullo*-4* |  | 0.0 | 22.0 |
| % diplo-*4* |  | 0.0 | 12.7 |

*a* Wild type = *y w; spa^pol^*.. *sina^A4^/sina^3^*= *y w/y; sina^A4^/sina^3^; spa^pol^.*

*b* NDJ, nondisjunction

*c* Adjusted Total is calculated to adjust for inviable progeny classes (see Methods).

**P<0.001 significantly different to wild type with the number of progeny scored.
